# Influent carbon to phosphorus ratio drives the selection of PHA-storing organisms in a single CSTR

**DOI:** 10.1016/j.wroa.2022.100150

**Published:** 2022-07-31

**Authors:** Antoine Brison, Pierre Rossi, Nicolas Derlon

**Affiliations:** a ETH Zürich, Institute of Environmental Engineering, Zürich 8093, Switzerland; b Eawag, Swiss Federal Institute of Aquatic Science and Technology, Dübendorf 8600, Switzerland; c Central Environmental Laboratory, School of Architecture, Civil and Environmental Engineering, Ecole Polytechnique Fédérale de Lausanne Lausanne, Switzerland

**Keywords:** Polyhydroxyalkanoates (PHAs), Dual carbon and phosphorus limitation, Growth conditions, Cellular phosphorus requirements, Biomass PHA content, Long-term selection

## Abstract

Enriching a biomass with a high fraction of polyhydroxyalkanoate-storing organisms (PHA-storers) represents an essential step in the production of PHAs (bioplastics) from municipal wastewater using mixed microbial cultures. A major challenge is however to create selective growth conditions that are favourable to PHA-storers. Our study thus investigates to what extent the influent COD to phosphorus (COD:P) ratio can be used as a tool for the robust selection of PHA-storers in a single continuous-flow stirred-tank reactor (CSTR). Therefore, we operated five CSTRs in parallel, fed with synthetic wastewater (50% acetate - 50% propionate) with different COD:P ratios (200−1000 gCOD gP^−1^), and performed a detailed analysis of the microbial communities over long-term (30−70 solid retention times). Our study demonstrates that efficient and robust selection of PHA-storers can be achieved in a single CSTR at high influent COD:P ratios. The selective advantage for PHA-storers increases with the influent COD:P ratio, but only if growth conditions remain limited by both C-substrate and P. In contrast, selection performance deteriorates when COD:P ratios are too high and growth conditions are limited by P only. At an optimal COD:P ratio of 800 gCOD gP^−1^, a stable microbial community consisting of >90% PHA-storers and dominated by *Pannonibacter* sp. was selected in the long-term. Finally, our results suggest that high COD:P ratios provide a selective advantage to microorganisms with low cellular P requirements, explaining why different PHA-storers (i.e., *Xanthobacter* sp*.* vs. *Pannonibacter* sp.*)* were selected depending on the influent COD:P ratio (i.e., 200 vs. 800 gCOD gP^−1^). Overall, our results provide relevant insights for the development of a new approach for selecting PHA-storers, based on the use of a single CSTR and control of the influent COD:P ratio.

## Highlights

• Selection of PHA-storers was studied in a single CSTR at high influent COD:P ratios.
• Stable microbial community with >90% PHA-storers at optimal COD:P (800 gCOD gP^−1^).
• Selective advantage for PHA-storers only when both C substrate and P limit growth.
• Low cellular P requirements provide selective advantage at high influent COD:P ratios.
• Pannonibacter *sp.* is a competitive PHA-storer at high influent COD:P ratios.

## Graphical abstract

**Figure:**
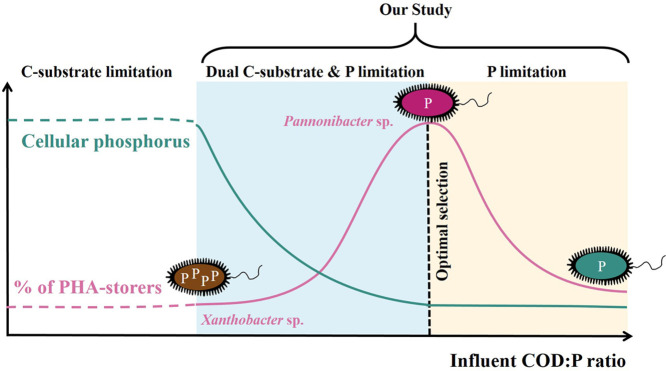

## 1 Introduction

The capture and side-stream anaerobic treatment of organic carbon (C), combined with mainstream anammox for nitrogen (N) removal, is a relevant treatment scheme for the wastewater treatment plant (WWTP) of the future (Alloul et al., 2018). In that context, volatile fatty acids (VFAs) produced *via* anaerobic fermentation of the organic C can be further upgraded into polyhydroxyalkanoates (PHAs) (bioplastics). An essential step in converting VFA-rich feedstock into PHAs consists in enriching a biomass with PHA-storing organisms (selection-step). While most WWTPs are operated in continuous-flow mode, the selection of PHA-storing organisms (PHA-storers) has been primarily investigated in discontinuous systems, i.e., in sequencing batch reactors (SBR) operated in an aerobic feast-famine regime (Estevez-Alonso et al., 2021; Reis et al., 2011; Valentino et al., 2017). A key challenge is therefore to be able to successfully select PHA-storers in continuous stirred tank reactors (CSTRs) as well, in order to gain more scope in the production of bioplastic from municipal wastewater (MWW) using mixed-microbial cultures. To date, however, the use of a simple CSTR for the selection-step has been hampered by our limited understanding of the environmental growth conditions that give a selective advantage to PHA-storers.

Very few studies investigated the selection of PHA-storers in CSTRs. Two CSTRs connected in series allow to impose an aerobic feast-famine regime on the biomass and, ultimately, to select PHA-storers (Albuquerque et al., 2010a; Bengtsson et al., 2008). Such a system however creates a weaker selective pressure than an SBR, since microbial competition is not only determined by the maximum rate of substrate uptake (as in a SBR), but also by substrate affinity (Marang et al., 2015; Paul et al., 2020). Another disadvantage of selecting PHA-storers by imposing feast-famine conditions in a continuous system is the need for two reactors. Phosphorus (P) limitation, however, could be a promising tool for selecting PHA-storers in a single CSTR. P-limiting conditions restrict the growth of heterotrophic bacteria while nutrient uptake capacity is maintained. Indeed, while growth is highly dependent on the synthesis of P-rich ribosomes and RNAs, the transmembrane proteins that control nutrient uptake in heterotrophic bacteria do not require P for their synthesis (Elser et al., 2003; Franklin et al., 2011). P-limiting conditions thus allow for excessive uptake of non-limiting C. The study of natural aquatic environments also suggests that accumulating excess C in form of intracellular storage compounds provides a competitive advantage to storing bacteria under P-limiting conditions (Ovreas et al., 2003; Thingstad et al., 2005). Further, strains of lake bacteria isolated in laboratory on P-poor media tended to accumulate C-rich compounds as opposed to strains of lake bacteria isolated on P-rich media (Godwin and Cotner, 2015, 2018). If the ability to store C provides a selective advandage in P-limited aquatic environments, an important question is to what extent P limitation (e.g., induced by high influent COD:P ratios) can be used in engineered systems to successfully select PHA-storers?

Influent COD:P ratios >270 gCOD gP^−1^ (i.e., 300 Cmol Pmol^−1^) hamper the selection of PHA-storers in aerobic feast-famine SBRs (Korkakaki et al., 2017), while dissolved COD:P ratios in the effluent of fermenters treating primary sludge can be as high as 1000 gCOD gP^−1^ (derived from Soares et al. (2010)). Yet, the use of high influent COD:P ratios as a tool for selecting PHA-storers in a single CSTR has not received much attention. In the only previous study, Cavaille et al. (2016) observed PHA contents up to ∼80 weight percent (wt%) while investigating how biomass responded to a stepwise increase of the influent COD:P ratio (80 – 2850 Cmol Pmol^−1^, corresponding to 80 – 2950 gCOD gP^−1^) as a function of solid retention time (SRT) (0.1–2 days). The authors noted that biomass PHA content increased with the influent COD:P ratio, and that higher SRTs were needed to prevent washout at higher COD:P ratios. Although the observations of Cavaille et al. (2016) suggest that high influent COD:P ratios select slow-growing PHA-storers in a single CSTR, additional evidence is required to better understand some key aspects of the selection process. Of particular interest are the relative abundance of PHA-storers in the biomass (efficiency of selection) and the stability of the PHA-storer community over the long-term (robustness of selection) when using C-substrates representative of MWW-derived feedstock. Cavaille et al. (2016) studied a simplified selective environment as they worked with acetate as the only C-substrate, neglecting the fact that MWW-derived feedstock also contains large amounts of propionate (Bahreini et al., 2020; Brison et al., 2022; Ucisik and Henze 2008). Furthermore, the above study did not examine to what extent the PHA-storer community increases and fluctuates in the long-term when the influent COD:P ratio is held constant. Yet, this robustness aspect is crucial for pratical application aiming at steady bioplastics production. To date, the link between the influent COD:P ratio and the selection of PHA-storers in a single CSTR remains poorly understood, primarily due to lack of detailed analysis of the microbial communities in such systems.

The present study therefore aims, for the first time, to understand the long-term selection (30-70 SRTs) of PHA-storers in a single CSTR at high influent COD:P ratios through a detailed analysis of the selected microbial communities. We therefore sought to answer the following questions: (i) Do high influent COD:P ratios allow to select a biomass with a large and stable PHA-storer community in the long-term? and (ii) What is the link between influent COD:P ratio, resulting growth conditions and selection of PHA-storers? To answer these questions, synthetic wastewaters (50% acetate – 50% propionate) with different COD:P ratios (200−1000 gCOD gP^−1^) were fed to five CSTRs running in parallel. Microbial communities, biomass PHA and P content, as well as COD-and PO_4_^3−^-P-removal were monitored over 30 to 70 SRTs.

## 2 Materials and methods

### 2.1 Experimental approach and synthetic wastewater composition

Five reactors with a working volume of 11 L were operated in parallel as single-stage CSTRs and fed with synthetic wastewater. COD:P ratios of 200, 400, 600, 800 and 1000 gCOD gP^−1^ were applied to the five reactors, respectively (Table 1). Influent COD concentration was kept constant at 8 gCOD L^−1^. The C-substrate consisted of 50% acetate and 50% propionate (COD basis). Acetate and propionate are often the most abundant VFAs in feedstock derived from the fermentation of MWW solids (e.g. primary sludge, sieved material) and were therefore used as C-substrates (Bahreini et al., 2020; Brison et al., 2022; Ucisik and Henze 2008). N in form of NH_4_Cl was dosed in excess and influent COD:N ratios ranged from 15-30 gCOD gN^−1^. C-substrate, N and P-species were added to the synthetic wastewater as indicated in Table 1. Further, the synthetic wastewater contained micro-nutrients (Ca^2+^, Mg^2+^, K^+^, SO_4_^2−^, Na^+^, Cl^−^) and trace elements in excess as specified in SI Table A1. To prevent microbial growth in the influent, the synthetic wastewater was prepared as two separate solutions: one containing the C-substrate and P species (and micro-nutrients), and a second one containing the N species (and trace elements). 10 L stock-solutions were prepared in 20-fold concentrations. Both stock-solutions, as well as distilled water for dilution, were continuously fed to the reactors *via* three automated peristaltic pumps. The stock-solutions were renewed every two to three weeks, while the reactor influent composition (COD, PO_4_^3−^-P and NH_4_^+^-N concentrations) was characterised 3 times a week to ensure a constant influent was maintained throughout the experiment. Also, the reactors were cleaned once a week to prevent biofilm growth.

**Table 1:** Target concentrations of C-substrate, N and P species in the synthetic wastewater, as well as measured influent parameters and operating conditions for the different reactors. When applicable: mean values ± standard deviations (number of measurements). Concerning the high NH_4_^+^-N concentrations in the influent, estimates of NH_3_-N bulk concentrations (always < 5mgN L^−1^) from NH_4_^+^-N measurements suggest NH_3_-N concentrations were not inhibiting heterotrophic growth (Cao et al., 2017).

CSTRs	C-substrate, N and P dosage					Reactor influent measurements		Operating conditions	
	Acetate	Propionate	N-species	P-species					
	NaC_2_H_3_O_2_	NaC_3_H_5_O_2_	NH_4_Cl	KH_2_PO_4_	K_2_HPO_4_	COD:P	sCOD	SRT = HRT	Duration
	[g L_influent_^−1^]	[g L_influent_^−1^]	[g L_influent_^−1^]	[g L_influent_^−1^]	[g L_influent_^−1^]	[gCOD gP^−1^]	[gCOD L^−1^]	[d]	[d]
COD:P 200	5.86	3.69	1.53	0.088	0.113	188 ± 7 (11)	8.4 ± 1.2 (11)	1.0 ± 0.1 (11)	30
COD:P 400	5.86	3.69	1.53	0.044	0.056	391 ± 15 (23)	8.1 ± 0.4 (23)	1.0 ± 0.0 (23)	57
COD:P 600	5.86	3.69	1.53	0.030	0.038	593 ± 20 (23)	8.5 ± 0.7 (23)	1.0 ± 0.1 (23)	57
COD:P 800	5.86	3.69	1.03	0.022	0.028	800 ± 26 (25)	8.0 ± 0.6 (25)	1.1 ± 0.0 (25)	70
COD:P 1000	5.86	3.69	1.03	0.018	0.023	992 ± 14 (25)	8.0 ± 0.4 (25)	1.0 ± 0.0 (25)	70

### 2.2 Operating conditions and detailed set-up

The different CSTRs were operated at a SRT of 1 day, and over a time-period of 30 to 70 days (Table 1). All reactors had a double-wall, and were equipped with temperature and pH sensors (Endress & Hauser, Switzerland). Temperature was controlled at 25 ± 1°C. pH was controlled at 7.5 through automated addition of a 3M HCl solution. Dissolved oxygen concentration was controlled at 3 mgO_2_ L^−1^. Sensors were connected to a programmable logic controller (PLC) and monitored by a supervisory control and data acquisition (SCADA) system. Mechanical stirrers were used for the mixing of the reactors. All reactors were inoculated with 4 mL of high-rate activated sludge from the Eawag wastewater treatment plant (Dübendorf, Switzerland). High-rate activated sludge was chosen as inoculum because the microorganisms therein (i) tend to have a high affinity towards intracellular storage of organic C, and (ii) are selected at similarly low SRTs (0.1−2 days) as in our experiments (Jimenez et al., 2015; Nogaj et al., 2019; Sancho et al., 2019).

### 2.3 Analytical methods

#### 2.3.1 Chemical analyses

Biomass samples were analysed for total COD, soluble COD (sCOD), total phosphorus (TP), ortho-phosphate (PO_4_^3−^-P), total nitrogen (TN) and ammonium nitrogen (NH_4_^+^-N) using colometric assays (Hach-Lange, Germany, LCK 014, 114, 303, 304, 338, 349, 350). sCOD, NH_4_^+^-N and PO_4_^3−^-P were measured after filtration at 0.45 µm (Macherey Nagel, Nanoclor Chromafil membranefilter GF/PET 0.45 µm, Germany). Particulate COD (pCOD) was calculated by subtracting the measured sCOD from the measured total COD. Samples were taken three times a week for COD and phosphorus species, and once a week for nitrogen species.

#### 2.3.2 PHA measurements

Biomass samples for PHA measurements were taken three times a week. Samples were immediately shock-frozen in liquid nitrogen to stop any biological activity and later stored at -18°C prior to lyophilisation. The lyophilised solids were then analysed for the most common PHA monomers produced by mixed microbial cultures from VFAs: 3HB, 3HV, 3H2MB and 3H2MV. PHA extraction, hydrolysis and analysis was performed as described in Lanham et al. (2013). Roughly 20 mg of lyophilised solids were mixed in a glass vial with 1 mL of acidified methanol (20% H_2_SO_4_) and 1 mL of chloroform containing 1 mg mL^−1^ of heptadecane (Sigma-Aldrich, Germany) as internal standard. Samples were then incubated at 100°C for 3.5 h for PHA extraction and further hydrolysed into its monomers. Ssamples were then cooled down on ice and vortexed for 1 min after adding 0.5 mL of nanopure water to aid phase separation. The lower phase (containing the chloroform) was transferred into 3 mL vials prior to analysis with a gas chromatograph coupled to a flame ionization detector (GC-FID) (Trace 1300 GC, Thermo Scientific, USA) and equipped with Zebron ZB-WAXplus (60 m x 0.53 mm x 1.00 µm) and Z-Guard (10 m x 0.32 mm) columns (both Phenomenex, USA). The detailed instrument method can be found in Lanham et al. (2013). A PHB-PHV co-polymer (86:14 weight %, Sigma-Aldrich) was used as a standard for 3HB and 3HV monomers. Industrial 3-hydroxy-2-methylbutanoic and 3-hydroxy-2-mehylpentanoic acids (both Merck, Germany) were used as standards for 3H2MB and 3H2MV monomers, respectively. As 3H2MB and 3H2MV were below the limit of quantification in all of the analysed samples, the total PHA concentration was calculated as the sum of PHB and PHV concentrations, deduced from the measured 3HB and 3HV signals. Results were expressed on a COD basis by using conversion factors of 1.67 gCOD gPHB^−1^ and 1.92 gCOD gPHV^−1^.

#### 2.3.3 Microbial community analysis

Biomass samples were collected twice a week for 16s rRNA gene sequencing. 1.5 mL of sludge were peletted at 12’000 × g for 5 min and washed twice in 3-4 mL ice-cold phosphate saline buffer (PBS). Pellets were homogenized with a glass homogenizer, and stored at -80°C until DNA extraction. DNA extraction and bacterial 16S rDNA amplicon sequencing was carried out as described in Layer et al. (2019) (Supporting Information A). The raw sequences are accessible under https://doi.org/10.25678/0006KT .

The definition of OTUs and the taxonomic affiliation was performed using FROGS pipeline (Escudie et al., 2018; Poirier et al., 2018). OTUs containing less than 0.01% of all sequences were excluded. Taxons were automatically affiliated in FROGS using 16S Silva 138 (Quast et al., 2013). The BLAST tool of the MiDAS Field Guide (https://www.midasfieldguide.org/guide/blast) was further used to improve affiliation at the genus/species taxonomic level of the 50 most abundant OTUs. The generated output including changes according to the MiDAS data base is provided in the Supporting Information B. The freeware R version 4.0.2 (R Core Team 2020) running on RStudio (version 1.3.1093) was used for numerical ecology analysis (Coral et al., 2018). Ruzicka dissimilarity matrices were calculated and visualized in corresponding color plots as described in Borcard et al. (2011). Principal component analysis (PCA) was carried out on Hellinger transformed relative abundancies of bacterial taxa using the Vegan package (Oksanen et al., 2020).

#### 2.3.4 Definition of steady-state

This study focused on the long-term effects of the influent COD:P ratio on the selection of PHA-storers. Only the steady-state period of each reactor was thus considered for the more detailed analysis of the microbial communities. Steady-state was defined as the time period during which the microbial community composition was in a dynamic equilibrium, i.e., where fluctuations were smallest and constant over time. This time period was determined for each reactor using a robust mathematical approach at the order taxonomic level:

1 A Ruzicka dissimilarity matrix was computed, expressing the dissimilarity between samples in terms of microbial community composition with a coefficient between 0 (identical) and 1 (highest dissimilarity possible) (SI Tables A2−A6).
2 An average dissimilarity coefficient was computed for each sample with respect to all subsequent samples. The average dissimilarity coefficient of the different samples was then plotted over time (Figs. SI A1–A5). Steady-state was visually defined as the time period where the average dissimilarity coefficient was not trending over time, indicating that the microbial communities were in a dynamic equilibrium. The absence of a trend during the selected steady-state period was confirmed with a Mann-Kendall test.
3 The steady-state period selected based on the Ruzicka dissimilarity matrix was further validated with a PCA-analysis by considering the sample clusters formed on the first two principal components (PC1 and PC2) (Fig. SI A6).

The definition of steady-state was done at the order taxonomic level for the following reasons: (i) more aggregated taxonomic levels (phylum, class) did not allow to define a clear temporal evolution of the microbial community in each reactor; (ii) at lower taxonomic levels, the microbial community in certain reactors hardly stabilized at all. The duration to reach steady-state as defined by the above criteria ranged from 7 to ∼40 days, depending on the COD:P ratios (SI Fig. A6).

#### 2.3.5 Identification of PHA-storers during steady-state

Microbial community analysis was performed at the genus taxonomic level, as at least such level of detail was required to verify whether individual bacterial taxa were potential PHA-storers. To this end, we conducted a detailed literature survey on 19 genera that were most abundant (average relative abundance >2% in at least one of the reactors during steady-state) and covered on average 83% ± 10%, 83% ± 9%, 92% ± 2%, 98% ± 2% and 94% ± 3% of the sequences in the COD:P 200–1000 reactors, respectively (SI Table A7). Information were obtained from *Bergey's manual of systematics of archea and bacteria* (https://onlinelibrary.wiley.com/doi/book/10.1002/9781118960608) and/or publications reporting a specific genus/species with PHA-storage function. A genus was considered a known PHA-storer when (i) literature clearly indicated that PHA-storage was a common trait to all species of that genus, or when (ii) affiliation at the species taxonomic level (polished with the MiDAS BLAST tool) allowed confirming that most sequences affiliated to that genus belonged to known PHA-storers. In contrast, a genus was considered a putative PHA-storer when at least two of its species were clearly identified as PHA-storers in the literature, but taxonomic affiliation at the species level in our study did not allow to clearly confirm the ability of the corresponding taxa to store PHA. When estimating the relative abundance of PHA-storers in the selected microbial communities both the known and the putative PHA-storers were considered.

### 2.4 Calculations

Average values for biomass P content, biomass PHA content, substrate to active biomass conversion yield and substrate to PHA conversion yields were calculated for the steady-state period only. The P content of the PHA-free biomass allows to estimate the cellular P requirements of the selected active biomass under the assumption that no other solids than active cells and PHA are formed (Cavaille et al., 2016; Korkakaki et al., 2017). The P content of the active biomass (biomass iP, in mgP gpCOD_PHA-free_^−1^) was calculated as in Korkakaki et al. (2017) and Cavaille et al. (2016):

(1) $$\text{Biomass}\;\text{iP}\;=\;\frac{P_{\text{org}}}{(\text{pCOD}\;-\;\text{CO}D_{\text{PHA}})}$$

With P_org_ the concentration of biomass-bound P (mgP L^−1^), pCOD the particulate COD concentration (gCOD L^−1^), and COD_PHA_ the PHA concentration in the reactor (gCOD_PHA_ L^−1^). P_org_ was calculated by subtracting measured PO_4_^3−^-P from measured TP.

The biomass PHA content (gCOD_PHA_ gpCOD^−1^) was calculated as:

(2) $$\text{PHA}\;\text{content}\;=\;\frac{\text{CO}D_{\text{PHA}}}{\text{pCOD}}$$

The substrate to active biomass conversion yield (active biomass yield, in gpCOD_PHA-free_ gCOD_removed_^−1^) was calculated as:

(3) $$\text{Active}\;\text{biomass}\;\text{yield}\;=\;\frac{(\text{pCOD}\;-\;\text{CO}D_{\text{PHA}})}{(\text{sCO}D_{\text{in}}\;-\;\text{sCO}D_{\text{eff}})}$$

With sCOD_in_ the influent sCOD (gCOD L^−1^), sCOD_eff_ the effluent sCOD (gCOD L^−1^), and with all of the influent COD (substrate) being in the dissolved form.

Similarily, the substrate to PHA conversion yield (PHA yield, in gpCOD_PHA_ gCOD_removed_^−1^) was calculated as:

(4) $$\text{PHA}\;\text{yield}\;=\;\frac{\;\text{CO}D_{\text{PHA}}}{(\text{sCO}D_{\text{in}}\;-\;\text{sCO}D_{\text{eff}})}$$

The raw data of all measured parameters is accessible under https://doi.org/10.25678/0006KT .

## 3 Results

### 3.1 How does the influent COD:P ratio influence the microbial community composition and the selection of PHA-storers?

**Fig. 1:**
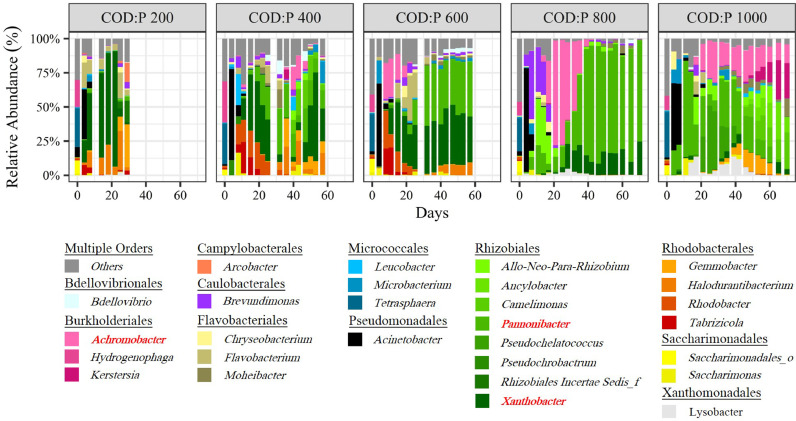
Microbial community composition over time in the different reactors at the taxonomic level of the genus. Genera are grouped at the order level. The group “Others” contains all taxa with a mean relative abundance <1% and a maximum relative abundance <10% with respect to all samples. The names of the three most abundant taxa were highlighted in bold-red (*Pannonibacter, Xanthobacter* and *Achromobacter*).

**Fig. 2:**
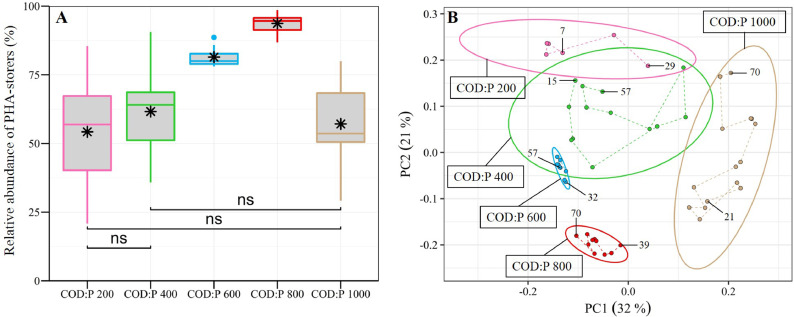
(A) Boxplot showing the relative abundance of PHA-storers in the selected microbial community during steady-state based on information gathered on the 19 most abundant genera (mean relative abundance of >2% in at least one of the reactors during steady-state) (see SI Table A7). The black stars in the boxes are the mean values. The symbols on top of the brackets indicate the statistical significance of the difference between mean values according to an Independent Samples t-test: ns (not significant, *p*>0.05), * (*p*<0.05), ** (*p*<0.01), *** (*p*<0.001) and **** (*p*<0.0001). (B) Principal Component Analysis (PCA) plot based on Hellinger transformed relative abundances of the bacterial taxa (genus level) present in the different reactors during steady-state. Samples (dots) appearing close to each other can be expected to be similar in terms microbial community composition. The dots corresponding to the onset of steady-state and the end of the experiment are labelled with the corresponding day. The ellipses cover the 90% confidence interval for each reactor, assuming a normal distribution. Thus, the ellipses are a visual indicator of the stability of the microbial community composition in the different reactors. PC3 (explaining 11% of the variance) vs PC1 and vs PC2 plots are provided in Supporting Information A (SI Fig. A7).

The microbial communities were monitored in the different reactors throughout the experiment (Fig. 1). The relative abundance of PHA-storers increased with the influent COD:P ratio from 200 to 800 gCOD gP^−1^ but then decreased when the COD:P ratio was further increased to 1000 gCOD gP^−1^ (Fig. 2A). Also, the relative abundance of PHA-storers as well as the overall microbial community composition were much more stable over time in the COD:P 600 and 800 reactors, as compared to the COD:P 200, 400 and 1000 reactors (Fig. 2A, B).

**Fig. 3:**
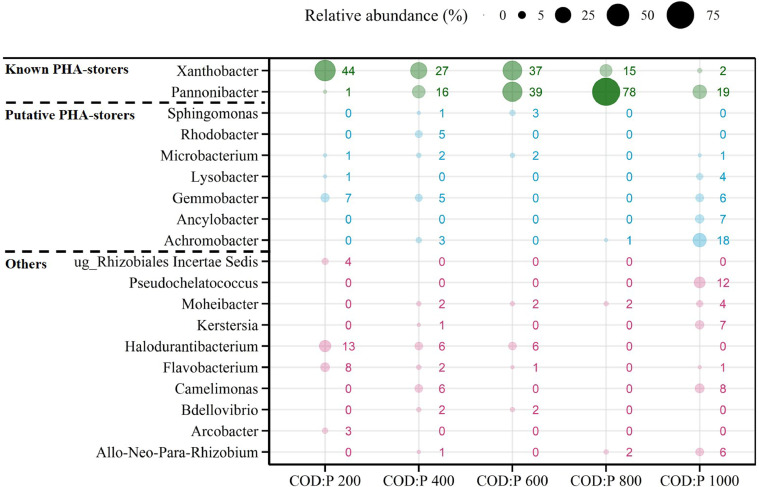
Balloon plot showing the average relative abundance of the 19 most abundant genera (average relative abundance >2% in at least one of the reactors during steady-state) in the COD:P 200-1000 reactors. The taxa are divided into three groups: the known PHA-storers (green), the putative PHA-storers (blue) and the others (red). The corresponding standard deviations and the literature references, based on which the genera were classified, are shown in SI Table A7.

The two most abundant genera across the different reactors, *Pannonibacter* and *Xanthobacter*, were confirmed to be known PHA-storers (Fig. 3). Concerning *Xanthobacter*, PHA-storage is a genus-wide feature (Wiegel, 2015). Concerning the *Pannonibacter* sp. found in the different reactors, 87-100% of the sequences were on average affiliated to *Pannonibacter phragmitetus*, which is a known PHA-storer (SI Table A7) (Borsodi et al., 2003; Ray et al., 2016). Also, *Pannonibacter* sp. are generally known to store PHA (Xi et al., 2018). Aside from *Xanthobacter* and *Pannonibacter* being confirmed as known PHA-storers, another 7 out of the 19 most abundant genera were identified as putative PHA-storers (Fig. 3). The average relative abundance of PHA-storers was quantified and found to be highest in the COD:P 800 reactor (94%, including 1% putative storers), followed by the COD:P 600 reactor (81%, including 5% putative storers) (Fig. 2A). Lower average relative abundancies of PHA-storers were observed at low COD:P ratios of 200 (54%, including 9% putative storers) and 400 gCOD P^−1^ (62%, including 17% putative storers) or at a very high COD:P ratio of 1000 gCOD P^−1^ (57%, including 36% putative storers). Further, the relative abundance of PHA-storers was most stable in the COD:P 600 and 800 reactors, with coefficients of variation of 4%. In contrast, much larger variations of the relative abundance of PHA-storers were observed in the COD:P 200, 400 and 1000 reactors, with coefficients of variation of 43%, 24% and 25%, respectively.

The influent COD:P ratio also influenced the composition of the PHA-storer community (Fig. 3). *Xanthobacter* sp. were the most abundant PHA-storers at a low COD:P ratio of 200 gCOD gP^−1^. But *Pannonibacter* sp. (*Pannonibacter phragmitetus*) outcompeted *Xanthobacter* sp. as the influent COD:P ratio increased from 200 to 800 gCOD gP^−1^: the relative abundance of *Xanthobacter* sp. decreased from 44% ± 34% to 15% ± 5%, while the relative abundance of *Pannonibacter* sp. continuously increased from 1% ± 1% up to 78% ± 6%. As the influent COD:P ratio was further increased to 1000 gCOD gP^−1^, the relative abundance of both *Pannonibacter* sp. and *Xanthobacter* sp. decreased down to 19% ± 12% and 2% ± 3% respectively, while in turn, the putative PHA-storer *Achromobacter* sp. proliferated (18% ± 6% of the sequences).

### 3.2 How does the influent COD:P ratio affect the biomass iP?

**Fig. 4:**
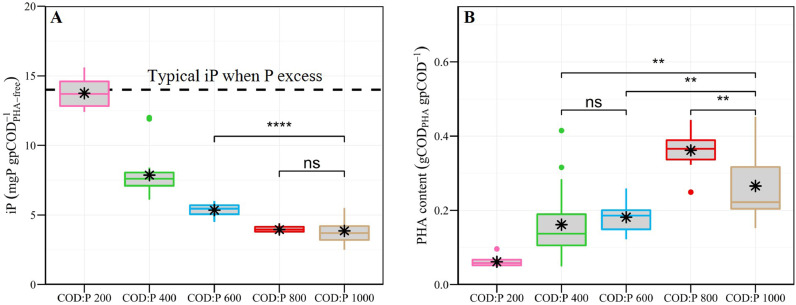
Boxplots showing the biomass iP (A) and PHA content (B) in the different reactors during steady-state. The black stars are mean values. The symbols on top of the brackets indicate the statistical significance of the differences between mean values: ns (not significant, *p*>0.05), * (*p*<0.05), ** (*p*<0.01), *** (*p*<0.001) and **** (*p*<0.0001).

The biomass iP was measured throughout the experiment in the different reactors (Figs. 4A, SI A8). In general, the higher the influent COD:P ratio, the lower the biomass iP (Fig. 4A). A reference iP-value of 14 mgP gpCOD_PHA-free_^−1^ can be assumed for bacterial cells when phosphorus is in excess (Tchobanoglous et al., 2013). A COD:P ratio of 200 gCOD gP^−1^ resulted in a similar biomass iP with an average value of 13.8 ± 1.1 mgP gpCOD_PHA-free_^−1^. The biomass iP then gradually decreased to 7.9 ± 1.6, 5.4 ± 0.5 and 4.0 ± 0.3 mgP gpCOD_PHA-free_^−1^ at COD:P ratios of 400, 600 and 800 gCOD gP^−1^, respectively. In the COD:P 1000 reactor, the biomass iP (3.9 ± 0.9 mgP gpCOD_PHA-free_^−1^) did not further diminish compared to the COD:P 800 reactor. In all reactors, the reduction of the biomass iP relative to a baseline of 14 mgP gpCOD_PHA-free_^−1^ occurred within the first day of the experiment (SI Fig. A8).

### 3.3 How does the influent COD: P ratio affect the PHA-storage response?

PHA content, PHA composition and substrate conversion yields were measured throughout the experiment in the different reactors (Figs. 4B, SI A9). Overall, higher influent COD:P ratios lead to a higher biomass PHA content (Fig. 4B). The average PHA content was highest in the COD:P 800 reactor (0.36 ± 0.05 gCOD_PHA_ gpCOD^−1^) followed by the COD:P 1000 reactor (0.27 ± 0.09 gCOD_PHA_ gpCOD^−1^), and lowest in the COD:P 200 reactor (0.06 ± 0.01 gCOD_PHA_ gpCOD^−1^). The COD:P 400 and 600 reactors had intermediary PHA contents of 0.16 ± 0.09 and 0.18 ± 0.04 gCOD_PHA_ gpCOD^−1^, respectively.

Furthermore, the more stable the PHA-storer community, the more stable the PHA-storage response: the biomass PHA content was therefore most stable in the COD:P 800 and 600 reactors, with coefficients of variation of 15% and 22%, respectively. In contrast, the greatest variations of PHA content were observed in the COD:P 400 and 1000 reactors (coefficients of variation of 57% and 36%, respectively). In all reactors, the biomass started to store PHA within the first 5 days of the experiment, well before the microbial community reached a dynamic equilibrium (SI Fig. A9). Overall, the monomeric composition of PHAs was similar in all reactors with primarily 3HB, small amounts of 3HV and negligible amounts of 3H2MB and 3H2MV (below limit of quantification). High fractions of 3HV (up to 50% of the PHA content) were observed only for the COD:P 400 reactor and occurred in parallel with sudden peaks of PHA content (SI Fig. A9).

Ultimately, the influent COD:P ratio also dictated to what extent C-substrate was utilized for the production of active biomass or for PHA-storage. Active biomass yields were lowest in the COD:P 800 and 600 reactors with 0.30 ± 0.03 and 0.31 ± 0.04 gpCOD_PHA-free_ gCOD_removed_^−1^, respectively. Higher active biomass yields were observed in the COD:P 200, 400 and 1000 reactors, with 0.36 ± 0.07, 0.35 ± 0.04 and 0.34 ± 0.07 gpCOD_PHA-free_ gCOD_removed_^−1^, respectively. Similarily than for the PHA content, PHA yield was highest in the COD:P 800 reactor (0.18 ± 0.03 gCOD_PHA_ gCOD_removed_^−1^), followed by the COD:P 1000 reactor (0.13 ± 0.03 gCOD_PHA_ gCOD_removed_^−1^), while it was lowest in the COD:P 200 reactor (0.02 ± 0.01 gCOD_PHA_ gCOD_removed_^−1^). Intermediary PHA yields were found for the COD:P 400 and 600 reactors: 0.07 ± 0.04 and 0.07 ± 0.01 gCOD_PHA_ gCOD_removed_^−1^, respectively.

### 3.4 How does the influent COD:P ratio affect COD-and PO_4_^3−^-P-removal?

**Fig. 5:**
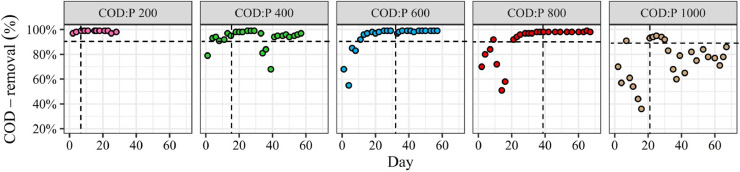
C-substrate (COD) removal over time in the different reactors. The vertical dashed line indicates the onset of steady-state with respect to the microbial community composition. The horizontal dashed line highlights the 90% removal benchmark. The reason for the sudden drop in COD-removal between day 35 and day 40 in the COD:P 400 reactor is unknown, but not related to operational problems.

COD-and PO_4_^3−^-P-removal performances were monitored throughout the experiment in the different reactors (Figs. 5, SI A10). Full and stable COD-removal (≥95%) was achieved in the COD:P 200-800 reactors. On the contrary, COD-removal in the COD:P 1000 reactor was unstable and fluctuated between 60 and 90% during the entire experiment. Also, the period to reach full COD-removal gradually increased from 1 day in the COD:P 200 reactor to 20 days in the COD:P 800 reactor. However, full COD-removal (when occuring) was always achieved before the microbial community reached a dynamic equilibrium. In all reactors, complete and stable PO_4_^3−^-P-removal occurred throughout the entire duration of the experiment (SI Fig. A10).

### 3.5 What is the link between influent COD:P ratio, selection of PHA-storers, biomass iP and PHA content?

**Fig. 6:**
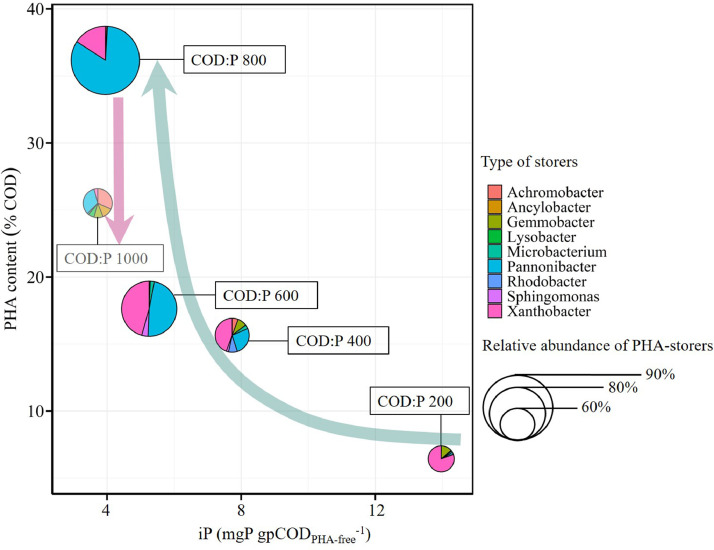
Scatter-pie plot showing the biomass PHA content (in %COD) as a function of the biomass iP and the selection of PHA-storers. The plot represents only average values from the steady-state. The center of the pie-charts marks the intersection of the biomass PHA content and iP. The size of the pie-charts is proportional to the relative abundance of PHA-storers within the selected microbial community. The pie-charts themselves specify the average composition of the PHA-storer community at the genus level. The COD:P 1000 reactor is “whitened” to highlight the partial COD removal. The arrows are intended to help read the plot. Dark-teal arrow: Increasing the COD:P ratio (200−800 gCOD gP^−1^) enhances the selection. Pink arrow: Increasing the COD:P ratio (800−1000 gCOD gP^−1^) mitigates the selection.

The results of the microbial community analysis were finally related to biomass iP and PHA content to gain further insight into the underlying relationships (Fig. 6). The selection of PHA-storers increased with the influent COD:P ratio between 200 and 800 gCOD gP^−1^, and coincided with increasing PHA-storage, reduction of the biomass iP, and full COD removal. As the COD:P ratio was further increased to 1000 gCOD gP^−1^, selection and COD removal performances deteriorated, while further reduction of the biomass iP was not observed. The influent COD:P ratio also governed the composition of the selected PHA-storer community, in particular the competition between gen. *Xanthobacter*, gen. *Pannonibacter* and gen. *Achromobacter*. On a more global scale, a loss in overall microbial diversity was observed in parallel to the reduction of the biomass iP for influent COD:P ratios >600 gCOD gP^-1^ (SI Fig. A11).

## 4 Discussion

### 4.1 High COD: P ratios allow to successfully select PHA-storers in a single CSTR

An efficient and robust selection process is characterised by a biomass with high and stable PHA-storage capacity. The PHA-storage capacity of a biomass in turn depends on both the relative abundance and the type of PHA-storers within the selected microbial community. An important question is then to what extent a single CSTR can be (i) efficient in terms of selecting a microbial community with a high relative abundance of PHA-storers, while being (ii) robust in terms of maintaining a large and stable PHA-storer community in the long-term?

Our results demonstrate a stable microbial community consisting of more than 90% PHA-storers can be successfully selected at a COD:P ratio of 800 gCOD gP^−1^ over a period of several weeks (Fig. 2A, B). Those results support the initial evidence by Cavaille et al. (2016) that PHA-storers can be selected in a CSTR at high COD:P ratios (up to 2950 gCOD gP^−1^) and at different SRTs (0.1–2 days). At similar operating conditions than in our study (COD:P ratio of ∼700 gCOD gP^−1^, SRT 1 day), Cavaille et al. (2016) also estimated the relative abundance of PHA-storers at >90%, based on the analysis of a limited number of biomass samples. The present study and the work of Cavaille et al. (2016) thus demonstrate that a single CSTR is as efficient in selecting PHA-storers as the state-of-the-art SBR (aerobic feast-famine) approach, where, for similar C substrates, the relative abundance of PHA-storers ranges typically between 70% and 95% (Albuquerque et al., 2013, 2010b; Lemos et al., 2008; Wang et al., 2017). Furthermore, for the first time, our study provides insights about the robustness of the selection process in a CSTR (using the example of the COD:P 800 reactor). First, we show that a high relative abundance of PHA-storers (>90%) can be steadily maintained over at least 30 SRTs (Figs. 1, 2A). Second, the microbial community that developed in the reactor was constantly dominated by the same PHA-storers *Pannonibacter* sp. (*Pannonibacter phragmitetus*) (78% ± 6%) and *Xanthobacter* sp. (15% ± 5%) (Borsodi et al., 2003; Ray et al., 2016; Wiegel, 2015), indicating a stable PHA-storer community developed over time (Fig. 1, SI Table A7). A stable PHA-storer community in turn translates into a stable PHA-storage capacity, implying a robust selection process. Such robustness is essential for scaling up PHA production using MWW-derived feedstock. However, the maximum PHA-storage capacity of the produced biomass ultimately depends on the type of PHA-storers selected. An important question is therefore to what extent different PHA-storers are selected in the single-stage CSTR (i) depending on the C-substrate, and (ii) compared to the SBR approach?

*Pannonibacter* sp. (*Pannonibacter phragmitetus*) (78% ± 6%) followed by *Xanthobacter* sp. (15% ± 5%) were the dominant PHA-storers selected on a synthetic wastewater composed of 50% acetate-propionate with a COD:P ratio of 800 gCOD gP^−1^ (Fig. 3). Different PHA-storers were selected by Cavaille et al. (2016) despite rather similar operating conditions (pH, temperature, COD:P ratio and SRT) while working with acetate as the sole C source: *Acidovorax* sp., *Brevundimonas* sp. and *Brachymonas* sp. Both *Brevundimonas* sp. and *Brachymonas* sp. are known to poorly grow on propionate (Hiraishi, 2015, Vancanneyt et al., 2015), which could explain why *Brevundimonas* sp. were washed out over time in our experiments, *e.g.*, in the COD:P 800 reactor (Fig. 1). The potential influence of the C-substrate composition on the competition among PHA-storers highlights the importance of working with VFA-mixtures that are relevant for practice, since the type of PHA-storers selected ultimately defines the maximum PHA-storage capacity of the biomass. The fermentation of MWW-derived solids (primary or activated sludge) typically yields a complex mixture of VFAs where both acetate and propionate are major constituents, together accounting for 70-90% of the VFAs, with the remainder consisting of (iso-)butyrate and (iso-)valerate (Brison et al., 2022; Da Ros et al., 2020; Ucisik and Henze, 2008). Working with a single C-substrate (e.g. acetate only) thus creates an oversimplified selective environment, poorly representative of real conditions. In contrast, the 50% acetate-propionate mix used in our study is more representative of the VFA mixture obtained from the fermentation of real MWW. Our results further suggest that for a similar C source, the CSTR approach selects different PHA-storers than the SBR approach. In SBR systems fed with acetate/propionate-rich feeds, the dominant PHA-storers typically belong to one of the following genera: *Zooglea, Thauera, Azoarcus, Amaricoccus, Plasticicumulans* and *Paracoccus* (Albuquerque et al., 2013, 2010b; Janarthanan et al., 2016; Jiang et al., 2011; Lemos et al., 2008; Morgan-Sagastume 2016; Wang et al., 2017). None of these SBR-typical PHA-storers dominated the microbial communities in our CSTR system, despite *Paracoccus* sp. and *Thauera* sp. being present in our inoculum.This indicates that either the high influent COD:P ratio or the associated lack of a substrate gradient (or a combination of both) provided a competitive advantage to other PHA-storers, such as *Pannonibacter* sp. (*Pannonibacter phragmitetus*) and *Xanthobacter* sp.. Interestingly, *Pannonibacter* sp. and *Xanthobacter* sp. were less abundant than *Paracoccus* sp. and *Thauera* sp. at the beginning of the experiments but successfully outcompeted them under the growth conditions in our CSTR system (SI Table A8) (Fig. 3). Korkakaki et al. (2017) observed how *Xanthobacter* sp. outcompeted *Plasticicumulans* sp. in an SBR, as they doubled the influent COD:P ratio (150 to 300 molC molP^−1^, corresponding to ∼140 to 270 gCOD gP^−1^). On the other side, Cavaille et al. (2016) observed that the SBR-typical *Zooglea* sp. dominated the microbial community in a CSTR (in absence of a substrate gradient), but only at rather low COD:P ratios (170−300 gCOD gP^−1^). We thus hypothesize the high influent COD:P ratio rather than the absence of a substrate gradient is responsible for the selection of different PHA-storers with the CSTR approach compared to the SBR approach, which is typically operated under nutrient excess, i.e., very low COD:N:P ratios (Valentino et al., 2017). Since different PHA-storers are selected with both approaches, future research should be directed towards investigating the maximum PHA-storage capacity of biomass selected with the CSTR approach, a key aspect not covered by our study.

### 4.2 Growth conditions and potential mechanisms behind the selection of PHA-storers

**Fig. 7:**
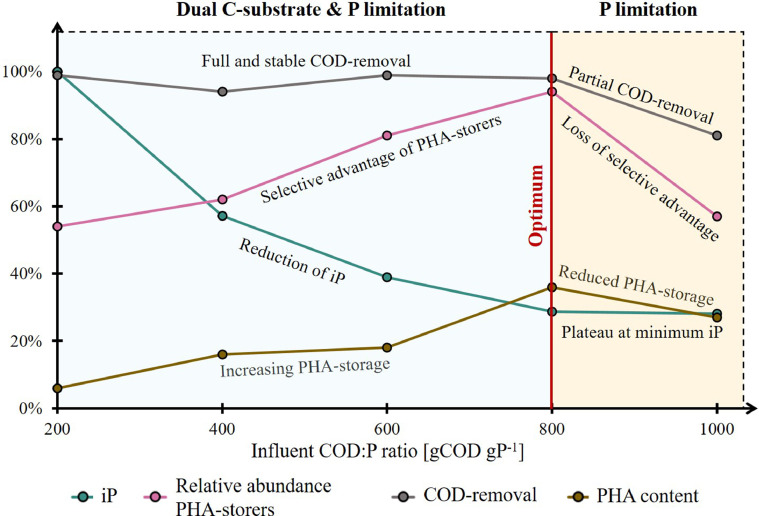
Conceptual summary of our results. Relative abundance of PHA-storers, biomass iP and PHA content, and COD-removal are represented on the y-axis in %. The reduction of the iP was expressed as the relative decrease of the biomass iP compared to the iP value measured in the COD:P 200 reactor. For each reactor, only average values are shown. The blue background indicates the range of COD:P ratios resulting in dual C-substrate and P-limited conditions, while the yellow background marks the beginning of the range of COD:P ratios leading to solely P-limited conditions.

Our results also clearly demonstrate that both efficiency and robustness of the selection process increase with the influent COD:P ratio from 200 to 800 gCOD gP^−1^, but then deteriorate when the ratio is further increased to 1000 gCOD gP^−1^ (Fig. 2A). If “too high” influent COD:P ratios mitigate selection performance, it is important to discuss the specific growth conditions under which PHA-storers have a selective advantage. In the present study, influent COD:P ratios between 200 and 800 gCOD gP^−1^ resulted in completely different growth conditions in the CSTRs than an influent COD:P ratio of 1000 gCOD gP^−1^. Both PO_4_^3−^-P and COD were fully consumed in the COD:P 200−800 reactors, whereas only partial COD-removal was observed in the COD:P 1000 reactor (Figs. 5, SI A10). Consequently, the growth environment in the COD:P 1000 reactor was characterized by an excess of C-substrate while P remained limiting. On the contrary, the environments in the COD:P 200-800 reactors were constantly depleted of both, C-substrate and P. Growth conditions where two nutrients are fully consumed at the same time have been described previously and referred to as dual nutrient-limited (Durner et al., 2000; Egli 1991; Grazerlampart et al., 1986; Zinn et al., 2004). A conceptual summary of our results reveals that the selection of PHA-storers improved together with an increasing influent COD:P ratio only as long as dual nutrient-limited conditions were maintained (Fig. 7). An important question is then (i) what mechanisms are involved in the selection of PHA-storers, and (ii) to what extent those mechanisms explain the existence of dual C-substrate and P-limited conditions over a certain range of influent COD:P ratios?

An increase in PHA-storage and a reduction in biomass iP occurred simultaneously with the selection of PHA-storers under dual C-substrate and P-limited conditions (Figs. 6, 7) (Cavaille et al., 2016). We hypothesize those two mechanisms (PHA-storage and reduction of biomass iP) were directly involved in the selection of PHA-storers when influent COD:P ratios increased from 200 to 800 gCOD gP^−1^. Under P-limited conditions, increasing cell size (and surface) *via* storage of non-limiting C-substrate might help microorganisms to maintain a high diffusive transport across the cell membrane and, ultimately, to maximize their affinity towards the limiting P (Ovreas et al., 2003; Thingstad et al., 2005). The growth conditions in the COD:P 200-800 reactors were by definition dual C-substrate and P-limited. However, as the COD:P ratios increased from 200 to 800 gCOD gP^−1^, less and less P was available for the formation of new cells. Such growth limitation typically results in more C-substrate being directed towards PHA synthesis in PHA-storers (Cavaille et al., 2016, 2013), explaining the higher PHA yields (and lower active biomass yields) in the COD:P 800 reactor (0.18 ± 0.03 gCOD_PHA_ gCOD_removed_^−^1) compared to the COD:P 200 reactor (0.02 ± 0.01 gCOD_PHA_ gCOD_removed_^−1^). Increased PHA-storage might in turn have allowed PHA-storers to increase their size and thus their affinity towards P, which could have ultimately helped them outcompete non-storing organisms. This potential PHA-storage related mechanism would however not explain the decreasing biomass iP (P content of PHA-free/active biomass) (Figs. 6, 7). Cavaille et al. (2016) hypothesized that reduction of biomass iP as a response to increasing COD:P ratios is due to the ability of the same microorganisms to adjust their cellular C:P ratio to ensure cell division (plasticity towards P). Our results however suggest that reduction of the biomass iP actually resulted from the selection of different microorganisms with low cellular P requirements rather than plasticity towards P. In the COD:P 800 reactor, the biomass iP was as low as 4 mgP gpCOD_PHA-free_^−1^ while *Pannonibacter* sp. accounted for ∼80% of the microbial community, indicating *Pannonibacter* sp. have low P requirements. Furthermore, increasing the influent COD:P ratio from 200 to 800 gCOD gP^−1^ caused a three-fold decrease of the biomass iP while microorganisms with low P requirements, such as *Pannonibacter* sp., gradually outcompeted microorganisms that coincided with high biomass iP, such as *Xanthobacter* sp. (Figs. 3, 6). This shift in microbial community composition, associated with a decrease of the biomass iP, is consistent with previous observations suggesting that low cellular P requirements provide a selective advantage when influent COD:P ratios are high (Godwin and Cotner, 2015). Overall, we hypothesize that under dual C-substrate and P-limited conditions, (i) the ability to store PHA drives competition between PHA-storers and non-storers, while (ii) the difference in cellular P requirements between microorganisms governs competition among PHA-storers (e.g. *Pannonibacter* sp. *vs. Xanthobacter* sp*.*).

No further reduction of the biomass iP was observed when the COD:P ratio was increased from 800 to 1000 gCOD gP^−1^, although very different microbial communities were selected (Figs. 2B, 6). These results indicate that the potential of a mixed culture to reduce its biomass iP *via* selection of microorganisms with low cellular P requirements is limited. The data also show that growth conditions change from dual C-substrate and P-limited to P-limited once this potential is exhausted (Fig. 7). Thus, the extent to which a mixed culture can reduce its biomass iP by selecting microorganisms with low cellular P requirements ultimately defines the range of influent COD:P ratios that will result in full removal of C-substrate and, thus, dual C-substrate and P-limited conditions. A main contribution of our study lies in identifying that selection of PHA-storers requires dual C-substrate and P-limited conditions combined with high influent COD:P ratios. Also, a conceptual summary is proposed (Fig. 7), based on which future research activities could be directed, including (i) confirming that cell size increase via PHA-storage is a key mechanism in the competition between PHA-storers and non-storers, as well as (ii) understanding why the selective advantage of PHA-storers is lost under solely P-limited conditions. Finally, the highest influent COD:P ratio at which dual C-substrate and P-limited conditions are maintained varies with operating conditions such as the SRT. The larger the SRT, the higher the COD:P ratio under which dual nutrient-limited conditions can be maintained. Cavaille et al. (2016) therefore observed dual C-substrate and P-limited conditions for COD:P ratios up to 2000 gCOD gP^−1^ at an SRT of 2 days, while at an SRT of 0.2 days COD:P ratios as low as 300 gCOD gP^−1^ were the limit for dual nutrient-limited growth. The same authors also observed that the PHA-storage capacity of the selected biomass increased with the COD:P ratio (80 – 2850 Cmol Pmol^−1^, corresponding to 80 – 2950 gCOD gP^−1^) and the SRT (0.1 – 2 days). An increasing SRT is however associated with decreasing biomass production (substrate to biomass conversion yields). Further research should be directed towards better understanding the growth of *Pannonibacter phragmitetus* under dual C-substrate and P-limited conditions in order to identify which operating conditions (COD:P ratio, SRT, etc…) allow to maximize both, the selective advantage for that PHA-storer and biomass production.

### 4.3 Practical implications

We demonstrate a microbial community dominated by PHA-storers can be successfully selected in a single CSTR fed with high influent COD:P ratios. Implementing such an approach in practice demands controlling the influent COD:P ratio. Reliable control of the influent COD:P ratio in turn requires online monitoring and chemical adjustment of the phosphorus concentration when needed. Since the COD:P ratios of MWW-derived feedstock range between 30 and 1000 gCOD gP^−1^ (Brison et al., 2022; Da Ros et al., 2020; Soares et al., 2010), precipitation of PO_4_^3−^-P might be required upfront the selection reactor. Chemical precipitation of PO_4_^3−^-P *via* metal salts (iron, alum, calcite/lime, etc…) can be easily implemented and has been widely applied in wastewater treatment over the past decades (de-Bashan and Bashan, 2004). The more challenging step in controlling the influent COD:P ratio will be the online monitoring of dissolved COD and PO_4_^3−^-P required to ensure the correct dosage of metal salts to the precipitation tank. Monitoring dissolved COD in the range of several 1000 mg L^−1^ (typical for MWW -derived feedstock) requires the use of UV/VIS spectrometry based sensors (e.g. s::can sensors) (Langergraber et al., 2004), while online monitoring of PO_4_^3−^-P can be done with ion selective sensors (e.g., based on molecular imprinted polymer) or colorimetric analyzers (Cornelissen et al., 2018; Warwick et al., 2014). Overall, implementing a functioning control of the influent COD:P ratio should not represent a critical technical bottleneck to the CSTR approach. Furthermore, the use of high influent COD:P ratios to valorize organic C in form of PHAs has the advantage of simultaneously encouraging plant operators to also remove P, another key nutrient in MWW that needs to be recovered and cycled back into society (Desmidt et al., 2015).

Finally, one must be careful when discussing the relevance of using a single CSTR for the production of PHAs (selection and accumulation) as opposed to using the state-of-the-art SBR (aerobic feast-famine). A proper evaluation of the CSTR would require a global comparison to the SBR, including a detailed evaluation of both the selection (storage capacity of the selected biomass, substrate to biomass conversion yields, robustness over time) and accumulation performances using the same real MWW-derived feedstock.

## 5 Conclusions

1 Efficient and robust selection of PHA-storers can be achieved in a single CSTR at high influent COD:P ratios. A stable microbial community consisting of >90% PHA-storers and dominated by *Pannonibacter* sp. (*Pannonibacter phragmitetus*) was selected in the long-term at an optimal influent COD:P ratio of 800 gCOD gP^-1^.
2 The selective advantage of PHA-storers over non-storing microorganisms increases with the influent COD:P ratio only as long as dual C-substrate and P-limited conditions prevail in the system (COD:P ratios 200-800 gCOD gP^−1^). Indeed, the selection performance deteriorates when the influent COD:P ratio is too high (1000 gCOD gP^−1^) and growth conditions become P-limited only.
3 Increased PHA-storage and reduction of biomass iP were observed to accompany selection of PHA-storers in dual C-substrate and P limited environments. First, we hypothesize PHA-storage provides a selective advantage by increasing cell size (and surface), ultimately allowing for a higher diffusive uptake of limiting P. Second, we demonstrate high COD:P ratios provide a selective advantage to microorganisms with low cellular P requirements, explaining why different PHA-storers were selected depending on the influent COD:P ratio (e.g., *Pannonibacter* sp. vs. *Xanthobacter* sp.).
4 Overall, a novel approach based on a single CSTR and controlling the influent COD:P ratio is proposed for the enrichment of a biomass with PHA-storers.

## Declaration of Competing Interest

The authors declare that they have no known competing financial interests or personal relationships that could have appeared to influence the work reported in this paper.

## Data Availability

A link to the raw data (with own DOI on an open repository) is provided in the manuscript. The DOI will be unlocked upon acceptance of the manuscript.

## Appendix Supplementary materials

Image, application 1

Image, application 2
