# Supplementary material for: Influent carbon to phosphorus ratio drives the selection of PHA-storing organisms in a single CSTR
Source: Water Res X. 2022 Jul 31;16:100150. doi: 10.1016/j.wroa.2022.100150 (PMC9364015; doi:10.1016/j.wroa.2022.100150)
Supplement: Supplementary file 1 [file mmc1.docx]

Influent carbon to phosphorus ratio drives the selection of PHA-storing organisms in a single CSTR

**SUPPLEMENTARY INFORMATION A**

Antoine Brison*,**, Pierre Rossi***, Nicolas Derlon*

* Eawag, Swiss Federal Institute of Aquatic Science and Technology, 8600 Dübendorf, Switzerland
** ETH Zürich, Institute of Environmental Engineering, 8093 Zürich, Switzerland
*** Central Environmental Laboratory, School of Architecture, Civil and Environmental Engineering, Ecole Polytechnique Fédérale de Lausanne Lausanne, Switzerland

Emails of the authors: [Antoine.brison@eawag.ch](mailto:Antoine.brison@eawag.ch)

[Pierre.rossi@epfl.ch](mailto:Pierre.rossi@epfl.ch)

[Nicolas.derlon@eawag.ch](mailto:Nicolas.derlon@eawag.ch)

Corresponding author: [Nicolas.derlon@eawag.ch](mailto:Nicolas.derlon@eawag.ch)

# Material and methods

## Micro-nutrients and trace element dosage

**SI Table A1:** Concentrations of micro-nutrients and trace elements in the synthetic wastewater (target concentrations in the reactor influent). The micro-nutrients were added in 20-fold concentration directly to the stock solutions. For the trace elements, a 1000-fold concentrated solution was prepared at pH 6 and added to the N-species stock solution at a volume ratio of 2%.

| **Micro-nutrients** | **Formula** | **Concentration (g/L)** |
| --- | --- | --- |
| Calcium Chloride monohydrate | CaCl_2_*1H_2_O | 0.045 |
| Magnesium Sulfate | MgSO_4_ | 0.055 |
| Potassium Chloride | KCl | 0.033 |
| Sodium Bicarbonate | NaHCO_3_ | 0.200 |
| **Trace-elements** | **Formula** | **Concentration (mg/L)** |
| EDTA disodium salt dihydrate | C_10_H_14_N_2_Na_2_O_8_*2H_2_O | 16.22 |
| Zinc II Sulfate | ZnSO_4_*7H_2_O | 0.44 |
| Manganese II Chloride | MnCl_2_*6H_2_O | 1.01 |
| Ammonium Iron II | (NH_4_)_2_Fe(SO_4_)_2_*6H_2_O | 7.05 |
| Ammonium Molybdate | (NH_4_)6Mo_7_O_24_*4H_2_O | 0.33 |
| Copper II Sulfate | CuSO_4_*5H_2_O | 0.31 |
| Cobalt II Chloride | CoCl_2_*6H_2_O | 0.32 |

## DNA extraction

200 μL of the homogenized biomass were mixed with 400 μL of TE buffer (10 mM Tris-HCl, 1 mM EDTA-Na_2_ pH 8.0) and 100 μL of lysozyme solution (25 mg mL^−1^), prior to incubation for 1 hour at 37 °C. DNA was extracted using the Maxwell® 16 robot and dedicated Tissue DNA purification kits (all Promega, USA) according to the manufacturer instructions. Quality measurement and quantification of the extracted DNA samples were assessed with agarose gels and fluorometric assays (Qubit ver. 2.0, Life Technologies, USA), respectively.

## 16S rRNA amplicon sequencing

Bacterial 16S rRNA gene hypervariable regions V1–V2 were amplified in a T3000 Thermocycler (Biometra, Germany) using 27F and 338R universal primers with overhang adapters (5’ TCGTCGGCAGCGTCAGATGTGTATAAGAGACAG-AGMGTTYGATYMTGGCTCAG3') and (5’GTCTCGTGGGCTCGGAGATGTGTATAAGAGACA-GGCTGCCTCCCGTAGGAGT3') (Layer et al. 2019). Amplification products were quantified on a Fragment Analyzer System with a NGS fragment kit (both Agilent, USA) prior to sequencing at the Lausanne Genomic Technologies Facility (University of Lausanne, Switzerland). Multiplex paired-end sequencing (2x250 bp) was carried out on an Illumina MiSeq platform.

## Definition of steady state

### COD:P 200 Reactor

**SI Table A2:** Ruzicka dissimilarity matrix for the COD:P 200 reactor and the corresponding average dissimilarity coefficients. The coefficients express the dissimilarity between samples in terms of microbial community composition with values ranging from 0 (identical) to 1 (highest dissimilarity possible) The black square highlights the defined period of dynamic equilibrium.

| Day | 0 | 1 | 4 | 7 | 14 | 18 | 22 | 25 | 29 | Average dissimilarity  coefficients |
| --- | --- | --- | --- | --- | --- | --- | --- | --- | --- | --- |
| 0 | 0.00 | 0.90 | 0.92 | 0.88 | 0.95 | 0.97 | 0.98 | 0.96 | 0.94 | 0.94 |
| 1 | 0.90 | 0.00 | 0.66 | 0.88 | 0.92 | 0.93 | 0.94 | 0.93 | 0.91 | 0.88 |
| 4 | 0.92 | 0.66 | 0.00 | 0.62 | 0.71 | 0.77 | 0.80 | 0.59 | 0.73 | 0.70 |
| 7 | 0.88 | 0.88 | 0.62 | 0.00 | 0.37 | 0.42 | 0.57 | 0.61 | 0.64 | 0.52 |
| 14 | 0.95 | 0.92 | 0.71 | 0.37 | 0.00 | 0.23 | 0.35 | 0.70 | 0.70 | 0.49 |
| 18 | 0.97 | 0.93 | 0.77 | 0.42 | 0.23 | 0.00 | 0.31 | 0.69 | 0.66 | 0.55 |
| 22 | 0.98 | 0.94 | 0.80 | 0.57 | 0.35 | 0.31 | 0.00 | 0.83 | 0.80 | 0.81 |
| 25 | 0.96 | 0.93 | 0.59 | 0.61 | 0.70 | 0.69 | 0.83 | 0.00 | 0.41 | 0.41 |
| 29 | 0.94 | 0.91 | 0.73 | 0.64 | 0.70 | 0.66 | 0.80 | 0.41 | 0.00 | - |

| 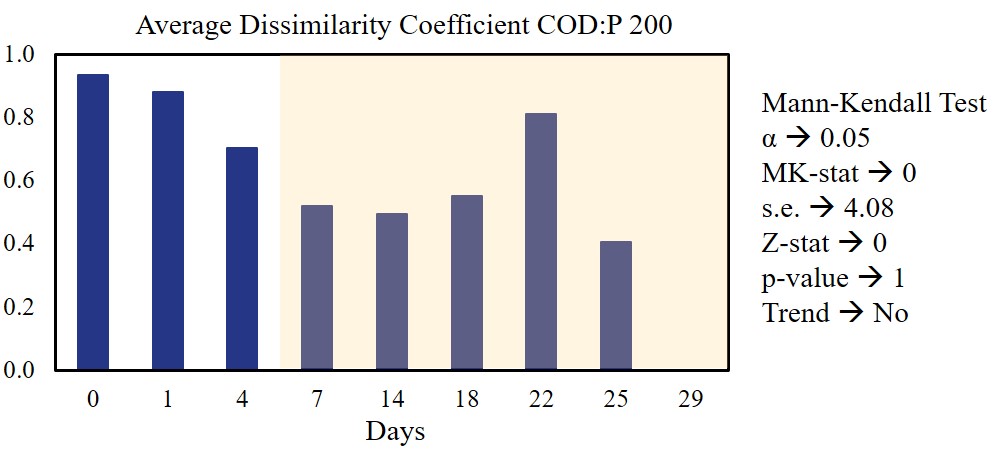  SI Figure A1: Average dissimilarity coefficient over time. The orange background indicates the visually selected steady-state, i.e. the period of dynamic equilibrium. The results of the Mann-Kendall Test for the selected period are shown on the right-hand side (confidence interval of 95%). |
| --- |

### COD:P 400 Reactor

**SI Table A3**: Ruzicka dissimilarity matrix for the COD:P 400 reactor and the corresponding average dissimilarity coefficients. The coefficients express the dissimilarity between samples in terms of microbial community composition with values ranging from 0 (identical) to 1 (highest dissimilarity possible) The black square highlights the defined period of dynamic equilibrium.

| Day | 0 | 1 | 4 | 8 | 11 | 15 | 19 | 22 | 25 | 32 | 36 | 40 | 43 | 47 | 50 | 53 | 55 | 57 | Average dissimilarity coefficients |
| --- | --- | --- | --- | --- | --- | --- | --- | --- | --- | --- | --- | --- | --- | --- | --- | --- | --- | --- | --- |
| 0 | 0.00 | 0.98 | 0.94 | 0.79 | 0.90 | 0.95 | 0.95 | 0.94 | 0.95 | 0.97 | 0.91 | 0.77 | 0.83 | 0.92 | 0.94 | 0.93 | 0.92 | 0.85 | 0.91 |
| 1 | 0.98 | 0.00 | 0.48 | 0.95 | 0.98 | 0.99 | 0.99 | 0.99 | 0.98 | 0.99 | 0.99 | 0.99 | 0.99 | 0.99 | 0.99 | 0.99 | 0.99 | 0.99 | 0.96 |
| 4 | 0.94 | 0.48 | 0.00 | 0.84 | 0.82 | 0.86 | 0.85 | 0.84 | 0.84 | 0.87 | 0.87 | 0.82 | 0.87 | 0.86 | 0.88 | 0.90 | 0.84 | 0.86 | 0.86 |
| 8 | 0.79 | 0.95 | 0.84 | 0.00 | 0.68 | 0.77 | 0.75 | 0.75 | 0.79 | 0.81 | 0.80 | 0.78 | 0.75 | 0.88 | 0.83 | 0.86 | 0.76 | 0.65 | 0.78 |
| 11 | 0.90 | 0.98 | 0.82 | 0.68 | 0.00 | 0.45 | 0.57 | 0.63 | 0.68 | 0.73 | 0.32 | 0.71 | 0.57 | 0.74 | 0.74 | 0.80 | 0.67 | 0.62 | 0.63 |
| 15 | 0.95 | 0.99 | 0.86 | 0.77 | 0.45 | 0.00 | 0.23 | 0.39 | 0.46 | 0.58 | 0.43 | 0.60 | 0.44 | 0.51 | 0.50 | 0.57 | 0.47 | 0.37 | 0.46 |
| 19 | 0.95 | 0.99 | 0.85 | 0.75 | 0.57 | 0.23 | 0.00 | 0.22 | 0.32 | 0.56 | 0.57 | 0.60 | 0.47 | 0.40 | 0.38 | 0.46 | 0.32 | 0.38 | 0.43 |
| 22 | 0.94 | 0.99 | 0.84 | 0.75 | 0.63 | 0.39 | 0.22 | 0.00 | 0.20 | 0.48 | 0.65 | 0.58 | 0.56 | 0.35 | 0.34 | 0.43 | 0.22 | 0.51 | 0.43 |
| 25 | 0.95 | 0.98 | 0.84 | 0.79 | 0.68 | 0.46 | 0.32 | 0.20 | 0.00 | 0.35 | 0.70 | 0.58 | 0.61 | 0.35 | 0.43 | 0.47 | 0.30 | 0.55 | 0.48 |
| 32 | 0.97 | 0.99 | 0.87 | 0.81 | 0.73 | 0.58 | 0.56 | 0.48 | 0.35 | 0.00 | 0.68 | 0.61 | 0.65 | 0.54 | 0.61 | 0.65 | 0.53 | 0.60 | 0.61 |
| 36 | 0.91 | 0.99 | 0.87 | 0.80 | 0.32 | 0.43 | 0.57 | 0.65 | 0.70 | 0.68 | 0.00 | 0.67 | 0.49 | 0.69 | 0.70 | 0.75 | 0.69 | 0.57 | 0.65 |
| 40 | 0.77 | 0.99 | 0.82 | 0.78 | 0.71 | 0.60 | 0.60 | 0.58 | 0.58 | 0.61 | 0.67 | 0.00 | 0.39 | 0.54 | 0.60 | 0.62 | 0.55 | 0.49 | 0.53 |
| 43 | 0.83 | 0.99 | 0.87 | 0.75 | 0.57 | 0.44 | 0.47 | 0.56 | 0.61 | 0.65 | 0.49 | 0.39 | 0.00 | 0.57 | 0.61 | 0.66 | 0.58 | 0.41 | 0.57 |
| 47 | 0.92 | 0.99 | 0.86 | 0.88 | 0.74 | 0.51 | 0.40 | 0.35 | 0.35 | 0.54 | 0.69 | 0.54 | 0.57 | 0.00 | 0.30 | 0.34 | 0.35 | 0.55 | 0.38 |
| 50 | 0.94 | 0.99 | 0.88 | 0.83 | 0.74 | 0.50 | 0.38 | 0.34 | 0.43 | 0.61 | 0.70 | 0.60 | 0.61 | 0.30 | 0.00 | 0.15 | 0.28 | 0.50 | 0.31 |
| 53 | 0.93 | 0.99 | 0.90 | 0.86 | 0.80 | 0.57 | 0.46 | 0.43 | 0.47 | 0.65 | 0.75 | 0.62 | 0.66 | 0.34 | 0.15 | 0.00 | 0.34 | 0.54 | 0.44 |
| 55 | 0.92 | 0.99 | 0.84 | 0.76 | 0.67 | 0.47 | 0.32 | 0.22 | 0.30 | 0.53 | 0.69 | 0.55 | 0.58 | 0.35 | 0.28 | 0.34 | 0.00 | 0.42 | 0.42 |
| 57 | 0.85 | 0.99 | 0.86 | 0.65 | 0.62 | 0.37 | 0.38 | 0.51 | 0.55 | 0.60 | 0.57 | 0.49 | 0.41 | 0.55 | 0.50 | 0.54 | 0.42 | 0.00 |  |

| 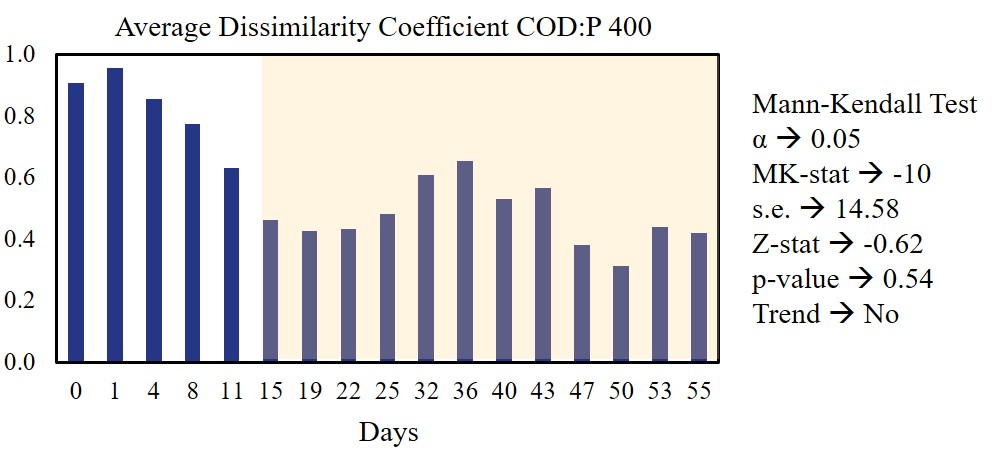  SI Figure A2: Average dissimilarity coefficient over time. The orange background indicates the visually selected steady-state, i.e. the period of dynamic equilibrium. The results of the Mann-Kendall Test for the selected period are shown on the right-hand side (confidence interval of 95%). |
| --- |

### COD:P 600 Reactor

**SI Table A4:** Ruzicka dissimilarity matrix for the COD:P 600 reactor and the corresponding average dissimilarity coefficients. The coefficients express the dissimilarity between samples in terms of microbial community composition with values ranging from 0 (identical) to 1 (highest dissimilarity possible) The black square highlights the defined period of dynamic equilibrium.

| Day | 0 | 1 | 4 | 8 | 11 | 15 | 19 | 22 | 25 | 32 | 36 | 40 | 43 | 47 | 50 | 53 | 55 | 57 | Average dissimilarity  coefficients |
| --- | --- | --- | --- | --- | --- | --- | --- | --- | --- | --- | --- | --- | --- | --- | --- | --- | --- | --- | --- |
| 0 | 0.00 | 0.89 | 0.83 | 0.75 | 0.76 | 0.82 | 0.88 | 0.91 | 0.92 | 0.97 | 0.94 | 0.90 | 0.93 | 0.93 | 0.93 | 0.93 | 0.92 | 0.92 | 0.89 |
| 1 | 0.89 | 0.00 | 0.52 | 0.87 | 0.88 | 0.89 | 0.92 | 0.96 | 0.96 | 0.97 | 0.96 | 0.96 | 0.97 | 0.97 | 0.98 | 0.97 | 0.97 | 0.97 | 0.92 |
| 4 | 0.83 | 0.52 | 0.00 | 0.94 | 0.94 | 0.96 | 0.95 | 0.95 | 0.93 | 0.96 | 0.94 | 0.92 | 0.95 | 0.95 | 0.95 | 0.95 | 0.95 | 0.94 | 0.95 |
| 8 | 0.75 | 0.87 | 0.94 | 0.00 | 0.46 | 0.65 | 0.80 | 0.86 | 0.89 | 0.94 | 0.92 | 0.91 | 0.91 | 0.91 | 0.92 | 0.90 | 0.91 | 0.89 | 0.85 |
| 11 | 0.76 | 0.88 | 0.94 | 0.46 | 0.00 | 0.44 | 0.64 | 0.72 | 0.75 | 0.79 | 0.77 | 0.74 | 0.73 | 0.74 | 0.74 | 0.72 | 0.74 | 0.71 | 0.71 |
| 15 | 0.82 | 0.89 | 0.96 | 0.65 | 0.44 | 0.00 | 0.36 | 0.54 | 0.54 | 0.65 | 0.64 | 0.61 | 0.61 | 0.61 | 0.61 | 0.59 | 0.61 | 0.57 | 0.58 |
| 19 | 0.88 | 0.92 | 0.95 | 0.80 | 0.64 | 0.36 | 0.00 | 0.36 | 0.35 | 0.60 | 0.57 | 0.53 | 0.54 | 0.54 | 0.54 | 0.52 | 0.53 | 0.51 | 0.51 |
| 22 | 0.91 | 0.96 | 0.95 | 0.86 | 0.72 | 0.54 | 0.36 | 0.00 | 0.44 | 0.69 | 0.66 | 0.63 | 0.64 | 0.65 | 0.64 | 0.62 | 0.65 | 0.63 | 0.62 |
| 25 | 0.92 | 0.96 | 0.93 | 0.89 | 0.75 | 0.54 | 0.35 | 0.44 | 0.00 | 0.44 | 0.43 | 0.34 | 0.44 | 0.47 | 0.47 | 0.45 | 0.47 | 0.45 | 0.44 |
| 32 | 0.97 | 0.97 | 0.96 | 0.94 | 0.79 | 0.65 | 0.60 | 0.69 | 0.44 | 0.00 | 0.12 | 0.25 | 0.23 | 0.25 | 0.27 | 0.25 | 0.24 | 0.28 | 0.24 |
| 36 | 0.94 | 0.96 | 0.94 | 0.92 | 0.77 | 0.64 | 0.57 | 0.66 | 0.43 | 0.12 | 0.00 | 0.21 | 0.19 | 0.19 | 0.21 | 0.19 | 0.19 | 0.22 | 0.20 |
| 40 | 0.90 | 0.96 | 0.92 | 0.91 | 0.74 | 0.61 | 0.53 | 0.63 | 0.34 | 0.25 | 0.21 | 0.00 | 0.16 | 0.20 | 0.20 | 0.18 | 0.19 | 0.16 | 0.18 |
| 43 | 0.93 | 0.97 | 0.95 | 0.91 | 0.73 | 0.61 | 0.54 | 0.64 | 0.44 | 0.23 | 0.19 | 0.16 | 0.00 | 0.07 | 0.08 | 0.08 | 0.09 | 0.10 | 0.08 |
| 47 | 0.93 | 0.97 | 0.95 | 0.91 | 0.74 | 0.61 | 0.54 | 0.65 | 0.47 | 0.25 | 0.19 | 0.20 | 0.07 | 0.00 | 0.05 | 0.07 | 0.05 | 0.10 | 0.07 |
| 50 | 0.93 | 0.98 | 0.95 | 0.92 | 0.74 | 0.61 | 0.54 | 0.64 | 0.47 | 0.27 | 0.21 | 0.20 | 0.08 | 0.05 | 0.00 | 0.05 | 0.07 | 0.10 | 0.07 |
| 53 | 0.93 | 0.97 | 0.95 | 0.90 | 0.72 | 0.59 | 0.52 | 0.62 | 0.45 | 0.25 | 0.19 | 0.18 | 0.08 | 0.07 | 0.05 | 0.00 | 0.09 | 0.08 | 0.08 |
| 55 | 0.92 | 0.97 | 0.95 | 0.91 | 0.74 | 0.61 | 0.53 | 0.65 | 0.47 | 0.24 | 0.19 | 0.19 | 0.09 | 0.05 | 0.07 | 0.09 | 0.00 | 0.09 | 0.09 |
| 57 | 0.92 | 0.97 | 0.94 | 0.89 | 0.71 | 0.57 | 0.51 | 0.63 | 0.45 | 0.28 | 0.22 | 0.16 | 0.10 | 0.10 | 0.10 | 0.08 | 0.09 | 0.00 | - |

| 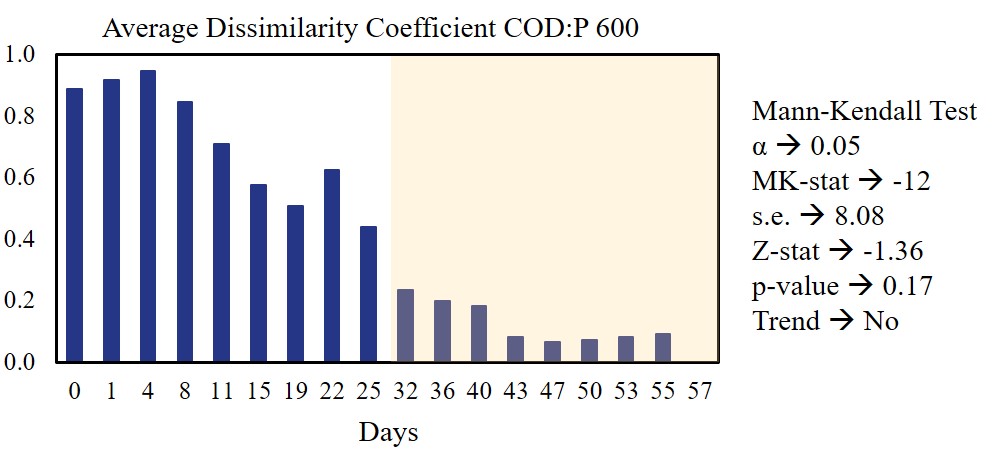  SI Figure A3: Average dissimilarity coefficient over time. The orange background indicates the visually selected steady-state, i.e. the period of dynamic equilibrium. The results of the Mann-Kendall Test for the selected period are shown on the right-hand side (confidence interval of 95%). |
| --- |

### COD:P 800 reactor

**SI Table A5**: Ruzicka dissimilarity matrix for the COD:P 800 reactor and the corresponding average dissimilarity coefficients. The coefficients express the dissimilarity between samples in terms of microbial community composition with values ranging from 0 (identical) to 1 (highest dissimilarity possible) The black square highlights the defined period of dynamic equilibrium.

| Day | 0 | 4 | 7 | 11 | 14 | 17 | 21 | 25 | 28 | 32 | 35 | 39 | 42 | 46 | 49 | 53 | 56 | 60 | 63 | 65 | 70 | Average dissimilarity coefficients |
| --- | --- | --- | --- | --- | --- | --- | --- | --- | --- | --- | --- | --- | --- | --- | --- | --- | --- | --- | --- | --- | --- | --- |
| 0 | 0.00 | 0.95 | 0.88 | 0.96 | 0.92 | 0.77 | 0.82 | 0.82 | 0.81 | 0.82 | 0.84 | 0.96 | 0.98 | 0.99 | 0.98 | 0.98 | 0.98 | 0.97 | 0.98 | 0.99 | 0.99 | 0.92 |
| 4 | 0.95 | 0.00 | 0.79 | 0.88 | 0.88 | 0.86 | 0.93 | 0.93 | 0.93 | 0.93 | 0.94 | 0.94 | 0.94 | 0.94 | 0.94 | 0.94 | 0.94 | 0.93 | 0.94 | 0.94 | 0.95 | 0.92 |
| 7 | 0.88 | 0.79 | 0.00 | 0.59 | 0.66 | 0.73 | 0.87 | 0.88 | 0.86 | 0.87 | 0.88 | 0.89 | 0.89 | 0.90 | 0.88 | 0.87 | 0.89 | 0.85 | 0.88 | 0.88 | 0.90 | 0.84 |
| 11 | 0.96 | 0.88 | 0.59 | 0.00 | 0.26 | 0.64 | 0.86 | 0.86 | 0.75 | 0.68 | 0.58 | 0.58 | 0.59 | 0.59 | 0.57 | 0.56 | 0.58 | 0.52 | 0.57 | 0.57 | 0.60 | 0.61 |
| 14 | 0.92 | 0.88 | 0.66 | 0.26 | 0.00 | 0.53 | 0.81 | 0.81 | 0.69 | 0.61 | 0.56 | 0.60 | 0.64 | 0.65 | 0.62 | 0.61 | 0.64 | 0.56 | 0.62 | 0.62 | 0.65 | 0.64 |
| 17 | 0.77 | 0.86 | 0.73 | 0.64 | 0.53 | 0.00 | 0.60 | 0.60 | 0.48 | 0.50 | 0.61 | 0.78 | 0.81 | 0.81 | 0.79 | 0.79 | 0.81 | 0.75 | 0.80 | 0.80 | 0.82 | 0.72 |
| 21 | 0.82 | 0.93 | 0.87 | 0.86 | 0.81 | 0.60 | 0.00 | 0.04 | 0.37 | 0.44 | 0.71 | 0.85 | 0.88 | 0.88 | 0.88 | 0.88 | 0.89 | 0.87 | 0.88 | 0.88 | 0.89 | 0.74 |
| 25 | 0.82 | 0.93 | 0.88 | 0.86 | 0.81 | 0.60 | 0.04 | 0.00 | 0.34 | 0.42 | 0.70 | 0.85 | 0.88 | 0.88 | 0.88 | 0.88 | 0.88 | 0.87 | 0.88 | 0.88 | 0.88 | 0.79 |
| 28 | 0.81 | 0.93 | 0.86 | 0.75 | 0.69 | 0.48 | 0.37 | 0.34 | 0.00 | 0.18 | 0.56 | 0.74 | 0.78 | 0.78 | 0.77 | 0.77 | 0.77 | 0.76 | 0.77 | 0.77 | 0.78 | 0.70 |
| 32 | 0.82 | 0.93 | 0.87 | 0.68 | 0.61 | 0.50 | 0.44 | 0.42 | 0.18 | 0.00 | 0.45 | 0.65 | 0.70 | 0.71 | 0.70 | 0.70 | 0.70 | 0.69 | 0.70 | 0.70 | 0.71 | 0.67 |
| 35 | 0.84 | 0.94 | 0.88 | 0.58 | 0.56 | 0.61 | 0.71 | 0.70 | 0.56 | 0.45 | 0.00 | 0.33 | 0.40 | 0.41 | 0.41 | 0.40 | 0.41 | 0.39 | 0.41 | 0.41 | 0.41 | 0.40 |
| 39 | 0.96 | 0.94 | 0.89 | 0.58 | 0.60 | 0.78 | 0.85 | 0.85 | 0.74 | 0.65 | 0.33 | 0.00 | 0.10 | 0.12 | 0.11 | 0.12 | 0.11 | 0.18 | 0.11 | 0.13 | 0.12 | 0.12 |
| 42 | 0.98 | 0.94 | 0.89 | 0.59 | 0.64 | 0.81 | 0.88 | 0.88 | 0.78 | 0.70 | 0.40 | 0.10 | 0.00 | 0.02 | 0.07 | 0.09 | 0.04 | 0.18 | 0.06 | 0.11 | 0.03 | 0.08 |
| 46 | 0.99 | 0.94 | 0.90 | 0.59 | 0.65 | 0.81 | 0.88 | 0.88 | 0.78 | 0.71 | 0.41 | 0.12 | 0.02 | 0.00 | 0.07 | 0.10 | 0.05 | 0.19 | 0.07 | 0.10 | 0.03 | 0.09 |
| 49 | 0.98 | 0.94 | 0.88 | 0.57 | 0.62 | 0.79 | 0.88 | 0.88 | 0.77 | 0.70 | 0.41 | 0.11 | 0.07 | 0.07 | 0.00 | 0.04 | 0.05 | 0.13 | 0.04 | 0.06 | 0.09 | 0.07 |
| 53 | 0.98 | 0.94 | 0.87 | 0.56 | 0.61 | 0.79 | 0.88 | 0.88 | 0.77 | 0.70 | 0.40 | 0.12 | 0.09 | 0.10 | 0.04 | 0.00 | 0.07 | 0.11 | 0.05 | 0.07 | 0.11 | 0.08 |
| 56 | 0.98 | 0.94 | 0.89 | 0.58 | 0.64 | 0.81 | 0.89 | 0.88 | 0.77 | 0.70 | 0.41 | 0.11 | 0.04 | 0.05 | 0.05 | 0.07 | 0.00 | 0.17 | 0.04 | 0.09 | 0.04 | 0.08 |
| 60 | 0.97 | 0.93 | 0.85 | 0.52 | 0.56 | 0.75 | 0.87 | 0.87 | 0.76 | 0.69 | 0.39 | 0.18 | 0.18 | 0.19 | 0.13 | 0.11 | 0.17 | 0.00 | 0.14 | 0.14 | 0.20 | 0.16 |
| 63 | 0.98 | 0.94 | 0.88 | 0.57 | 0.62 | 0.80 | 0.88 | 0.88 | 0.77 | 0.70 | 0.41 | 0.11 | 0.06 | 0.07 | 0.04 | 0.05 | 0.04 | 0.14 | 0.00 | 0.06 | 0.07 | 0.07 |
| 65 | 0.99 | 0.94 | 0.88 | 0.57 | 0.62 | 0.80 | 0.88 | 0.88 | 0.77 | 0.70 | 0.41 | 0.13 | 0.11 | 0.10 | 0.06 | 0.07 | 0.09 | 0.14 | 0.06 | 0.00 | 0.12 | 0.12 |
| 70 | 0.99 | 0.95 | 0.90 | 0.60 | 0.65 | 0.82 | 0.89 | 0.88 | 0.78 | 0.71 | 0.41 | 0.12 | 0.03 | 0.03 | 0.09 | 0.11 | 0.04 | 0.20 | 0.07 | 0.12 | 0.00 | - |

| 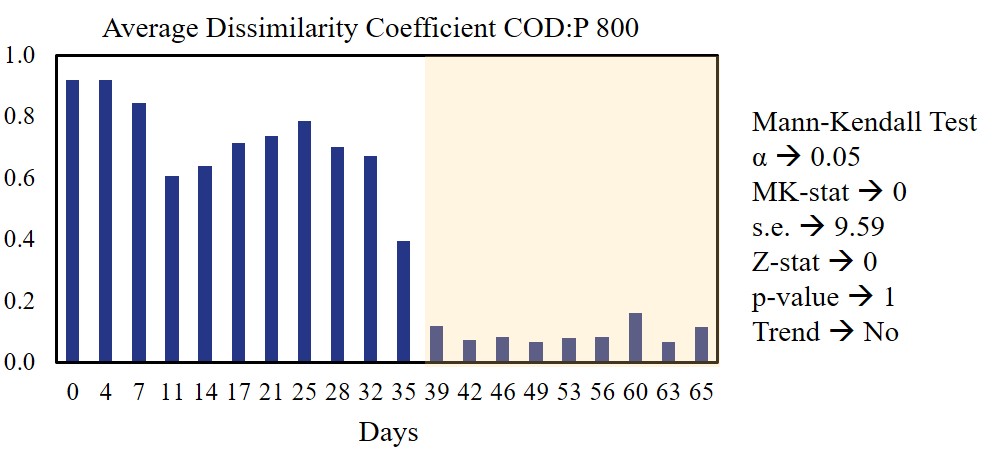  SI Figure A4: Average dissimilarity coefficient over time. The orange background indicates the visually selected steady-state, i.e. the period of dynamic equilibrium. The results of the Mann-Kendall Test for the selected period are shown on the right-hand side (confidence interval of 95%). |
| --- |

### COD:P 1000 reactor

**SI Table A6:** Ruzicka dissimilarity matrix for the COD:P 1000 reactor and the corresponding average dissimilarity coefficients. The coefficients express the dissimilarity between samples in terms of microbial community composition with values ranging from 0 (identical) to 1 (highest dissimilarity possible) The black square highlights the defined period of dynamic equilibrium.

| Day | 0 | 4 | 7 | 11 | 14 | 17 | 21 | 25 | 28 | 32 | 35 | 39 | 42 | 46 | 49 | 53 | 56 | 60 | 64 | 65 | 70 | Average dissimilarity coefficients |
| --- | --- | --- | --- | --- | --- | --- | --- | --- | --- | --- | --- | --- | --- | --- | --- | --- | --- | --- | --- | --- | --- | --- |
| 0 | 0.00 | 0.93 | 0.86 | 0.92 | 0.97 | 0.93 | 0.84 | 0.82 | 0.80 | 0.85 | 0.82 | 0.84 | 0.78 | 0.78 | 0.79 | 0.77 | 0.77 | 0.77 | 0.80 | 0.81 | 0.82 | 0.83 |
| 4 | 0.93 | 0.00 | 0.24 | 0.76 | 0.80 | 0.74 | 0.76 | 0.84 | 0.83 | 0.80 | 0.77 | 0.81 | 0.78 | 0.78 | 0.78 | 0.78 | 0.82 | 0.83 | 0.83 | 0.83 | 0.75 | 0.76 |
| 7 | 0.86 | 0.24 | 0.00 | 0.77 | 0.78 | 0.80 | 0.78 | 0.85 | 0.83 | 0.80 | 0.77 | 0.82 | 0.80 | 0.79 | 0.79 | 0.79 | 0.82 | 0.83 | 0.83 | 0.84 | 0.81 | 0.81 |
| 11 | 0.92 | 0.76 | 0.77 | 0.00 | 0.32 | 0.61 | 0.40 | 0.43 | 0.49 | 0.44 | 0.49 | 0.54 | 0.60 | 0.63 | 0.57 | 0.58 | 0.55 | 0.54 | 0.45 | 0.62 | 0.66 | 0.53 |
| 14 | 0.97 | 0.80 | 0.78 | 0.32 | 0.00 | 0.53 | 0.43 | 0.44 | 0.48 | 0.37 | 0.41 | 0.43 | 0.54 | 0.60 | 0.58 | 0.60 | 0.56 | 0.54 | 0.46 | 0.61 | 0.67 | 0.51 |
| 17 | 0.93 | 0.74 | 0.80 | 0.61 | 0.53 | 0.00 | 0.49 | 0.62 | 0.59 | 0.51 | 0.50 | 0.44 | 0.42 | 0.49 | 0.53 | 0.55 | 0.60 | 0.60 | 0.61 | 0.61 | 0.50 | 0.54 |
| 21 | 0.84 | 0.76 | 0.78 | 0.40 | 0.43 | 0.49 | 0.00 | 0.24 | 0.21 | 0.23 | 0.30 | 0.34 | 0.38 | 0.39 | 0.31 | 0.34 | 0.30 | 0.26 | 0.22 | 0.37 | 0.43 | 0.31 |
| 25 | 0.82 | 0.84 | 0.85 | 0.43 | 0.44 | 0.62 | 0.24 | 0.00 | 0.13 | 0.27 | 0.37 | 0.41 | 0.48 | 0.48 | 0.40 | 0.37 | 0.27 | 0.20 | 0.15 | 0.31 | 0.45 | 0.33 |
| 28 | 0.80 | 0.83 | 0.83 | 0.49 | 0.48 | 0.59 | 0.21 | 0.13 | 0.00 | 0.22 | 0.30 | 0.37 | 0.44 | 0.42 | 0.34 | 0.34 | 0.25 | 0.19 | 0.19 | 0.28 | 0.43 | 0.31 |
| 32 | 0.85 | 0.80 | 0.80 | 0.44 | 0.37 | 0.51 | 0.23 | 0.27 | 0.22 | 0.00 | 0.13 | 0.24 | 0.39 | 0.40 | 0.37 | 0.41 | 0.33 | 0.30 | 0.22 | 0.42 | 0.54 | 0.34 |
| 35 | 0.82 | 0.77 | 0.77 | 0.49 | 0.41 | 0.50 | 0.30 | 0.37 | 0.30 | 0.13 | 0.00 | 0.22 | 0.38 | 0.40 | 0.37 | 0.41 | 0.33 | 0.31 | 0.31 | 0.44 | 0.55 | 0.37 |
| 39 | 0.84 | 0.81 | 0.82 | 0.54 | 0.43 | 0.44 | 0.34 | 0.41 | 0.37 | 0.24 | 0.22 | 0.00 | 0.27 | 0.36 | 0.35 | 0.38 | 0.32 | 0.33 | 0.33 | 0.42 | 0.53 | 0.36 |
| 42 | 0.78 | 0.78 | 0.80 | 0.60 | 0.54 | 0.42 | 0.38 | 0.48 | 0.44 | 0.39 | 0.38 | 0.27 | 0.00 | 0.19 | 0.25 | 0.29 | 0.35 | 0.36 | 0.39 | 0.47 | 0.42 | 0.34 |
| 46 | 0.78 | 0.78 | 0.79 | 0.63 | 0.60 | 0.49 | 0.39 | 0.48 | 0.42 | 0.40 | 0.40 | 0.36 | 0.19 | 0.00 | 0.14 | 0.19 | 0.29 | 0.35 | 0.39 | 0.46 | 0.41 | 0.32 |
| 49 | 0.79 | 0.78 | 0.79 | 0.57 | 0.58 | 0.53 | 0.31 | 0.40 | 0.34 | 0.37 | 0.37 | 0.35 | 0.25 | 0.14 | 0.00 | 0.06 | 0.19 | 0.25 | 0.30 | 0.38 | 0.42 | 0.27 |
| 53 | 0.77 | 0.78 | 0.79 | 0.58 | 0.60 | 0.55 | 0.34 | 0.37 | 0.34 | 0.41 | 0.41 | 0.38 | 0.29 | 0.19 | 0.06 | 0.00 | 0.15 | 0.22 | 0.30 | 0.35 | 0.39 | 0.28 |
| 56 | 0.77 | 0.82 | 0.82 | 0.55 | 0.56 | 0.60 | 0.30 | 0.27 | 0.25 | 0.33 | 0.33 | 0.32 | 0.35 | 0.29 | 0.19 | 0.15 | 0.00 | 0.09 | 0.19 | 0.30 | 0.43 | 0.25 |
| 60 | 0.77 | 0.83 | 0.83 | 0.54 | 0.54 | 0.60 | 0.26 | 0.20 | 0.19 | 0.30 | 0.31 | 0.33 | 0.36 | 0.35 | 0.25 | 0.22 | 0.09 | 0.00 | 0.15 | 0.28 | 0.42 | 0.28 |
| 63 | 0.80 | 0.83 | 0.83 | 0.45 | 0.46 | 0.61 | 0.22 | 0.15 | 0.19 | 0.22 | 0.31 | 0.33 | 0.39 | 0.39 | 0.30 | 0.30 | 0.19 | 0.15 | 0.00 | 0.34 | 0.48 | 0.41 |
| 65 | 0.81 | 0.83 | 0.84 | 0.62 | 0.61 | 0.61 | 0.37 | 0.31 | 0.28 | 0.42 | 0.44 | 0.42 | 0.47 | 0.46 | 0.38 | 0.35 | 0.30 | 0.28 | 0.34 | 0.00 | 0.23 | 0.23 |
| 70 | 0.82 | 0.75 | 0.81 | 0.66 | 0.67 | 0.50 | 0.43 | 0.45 | 0.43 | 0.54 | 0.55 | 0.53 | 0.42 | 0.41 | 0.42 | 0.39 | 0.43 | 0.42 | 0.48 | 0.23 | 0.00 | - |

| 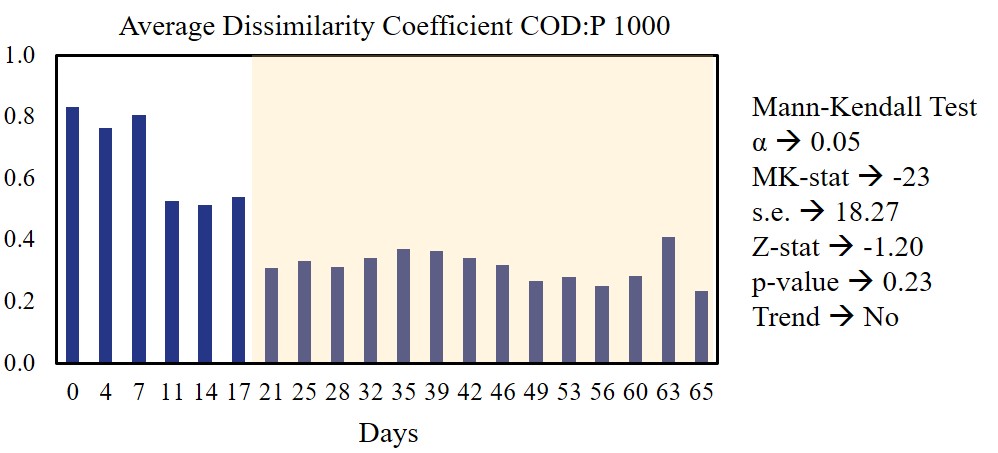  SI Figure A5: Average dissimilarity coefficient over time. The orange background indicates the visually selected steady-state, i.e. the period of dynamic equilibrium. The results of the Mann-Kendall Test for the selected period are shown on the right-hand side (confidence interval of 95%). |
| --- |

**Validation of steady state via PCA**

##
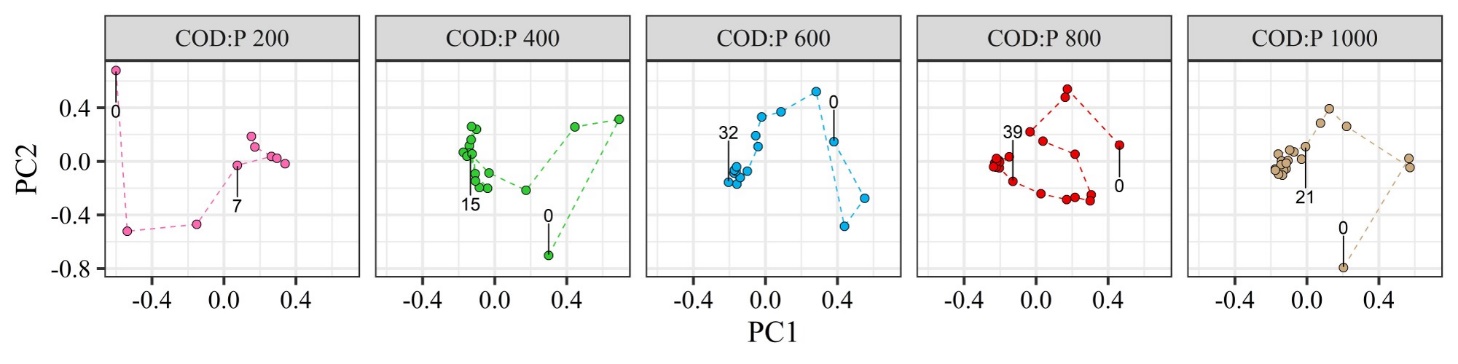


**SI Figure A6:** Plot of the principal component analyses (PCA) performed for each reactor individually with Hellinger-transformed relative abundances of the bacterial taxa (genus level). The inoculum and the first sample of the steady-state are in addition labeled with the respective experimental day.

## Identification of PHA-storers during steady-state

**SI Table A7**: Literature survey conducted to identify PHA-storers among the important 19 most abundant genera (average relative abundance >2% during steady-state) across the five reactors. The table shows average values ± standard deviation of the relative abundance. For the genus Pannonibacter, the value in parenthesis indicate how many of the sequences were affiliated to Pannonibacter Phragmitetus, a known PHA-storer. “-“ in the references field indicates that no references were found that mentioned the respective genus in relation with PHA storage.

|  | Average relative abundance during steady-state (%) | | | | |  |  |
| --- | --- | --- | --- | --- | --- | --- | --- |
| Genus | COD:P 200 | COD:P 400 | COD:P 600 | COD:P 800 | COD:P 1000 | Classification of the genus | References |
| *Xanthobacter* | 44 ± 34 | 27 ± 20 | 37 ± 4 | 15 ± 5 | 2 ± 3 | Confirmed PHA-storer | Wiegel (2015) |
| *Pannonibacter*  *(Pannonibacter phragmitetus)* | 1 ± 1 (98 ± 1) | 16 ± 9  (93 ± 5) | 39 ± 3  (98 ± 1) | 78 ± 6  (100 ± 0) | 19 ± 12  (87 ± 7) | Confirmed PHA-storer | Borsodi et al. (2003), Xi et al. (2018), Ray et al. (2016) |
| *Sphingomonas* | 0 ± 0 | 1 ± 3 | 3 ± 2 | 0 ± 0 | 0 ± 0 | Putative PHA-storer | Sood et al. (2022) |
| *Rhodobacter* | 0 ± 0 | 5 ± 8 | 0 ± 0 | 0 ± 0 | 0 ± 0 | Putative PHA-storer | Imhoff (2015) |
| *Microbacterium* | 1 ± 2 | 2 ± 5 | 2 ± 1 | 0 ± 0 | 1 ± 3 | Putative PHA-storer | Kumar et al. (2018), Li and Wilkins (2020) |
| *Lysobacter* | 1 ± 1 | 0 ± 0 | 0 ± 0 | 0 ± 0 | 3 ± 5 | Putative PHA-storer | Gasser et al. (2009), Von Tigerstrom and Stelmaschuk (1985) |
| *Gemmobacter* | 7 ± 13 | 5 ± 6 | 0 ± 0 | 0 ± 0 | 0 ± 0 | Putative PHA-storer | Sheu et al. (2013a), Sheu et al. (2013b) |
| *Ancylobacter* | 0 ± 0 | 0 ± 0 | 0 ± 0 | 0 ± 0 | 7 ± 9 | Putative PHA-storer | Xin et al. (2006), Xin et al. (2004), Tyagi and Sharma (2021) |
| *Achromobacter* | 0 ± 0 | 3 ± 6 | 0 ± 0 | 1 ± 2 | 18 ± 6 | Putative PHA-storer | Clifton‐García et al. (2020), Ferreira et al. (2016), Liu et al. (2018) |
| *ug_Rhizobiales Incertae Sedis* | 4 ± 6 | 0 ± 0 | 0 ± 0 | 0 ± 0 | 0 ± 0 | Not determined | - |
| *Pseudochelatococcus* | 0 ± 0 | 0 ± 0 | 0 ± 0 | 0 ± 0 | 12 ± 17 | Not determined | - |
| *Moheibacter* | 0 ± 0 | 2 ± 4 | 2 ± 1 | 2 ± 2 | 4 ± 4 | Not determined | - |
| *Kerstersia* | 0 ± 0 | 1 ± 2 | 0 ± 0 | 0 ± 0 | 7 ± 10 | Not determined | - |
| *Halodurantibacterium* | 13 ± 10 | 6 ± 7 | 6 ± 3 | 0 ± 0 | 0 ± 0 | Not determined | - |
| *Flavobacterium* | 8 ± 6 | 2 ± 4 | 1 ± 2 | 0 ± 0 | 1 ± 2 | Not determined | Holmes et al. (1982) |
| *Camelimonas* | 0 ± 0 | 6 ± 8 | 0 ± 0 | 0 ± 0 | 8 ± 8 | Not determined | Marín and Arahal (2014) |
| *Bdellovibrio* | 0 ± 0 | 2 ± 3 | 2 ± 1 | 0 ± 1 | 0 ± 0 | No | Martinez et al. (2012) |
| *Arcobacter* | 3 ± 6 | 0 ± 0 | 0 ± 0 | 0 ± 0 | 0 ± 0 | Not determined | Liu et al. (2013) |
| *Allo-Neo-Para-Rhizobium* | 0 ± 0 | 1 ± 2 | 0 ± 0 | 2 ± 1 | 6 ± 5 | Not determined | - |

# Results

## Overall microbial community composition

| 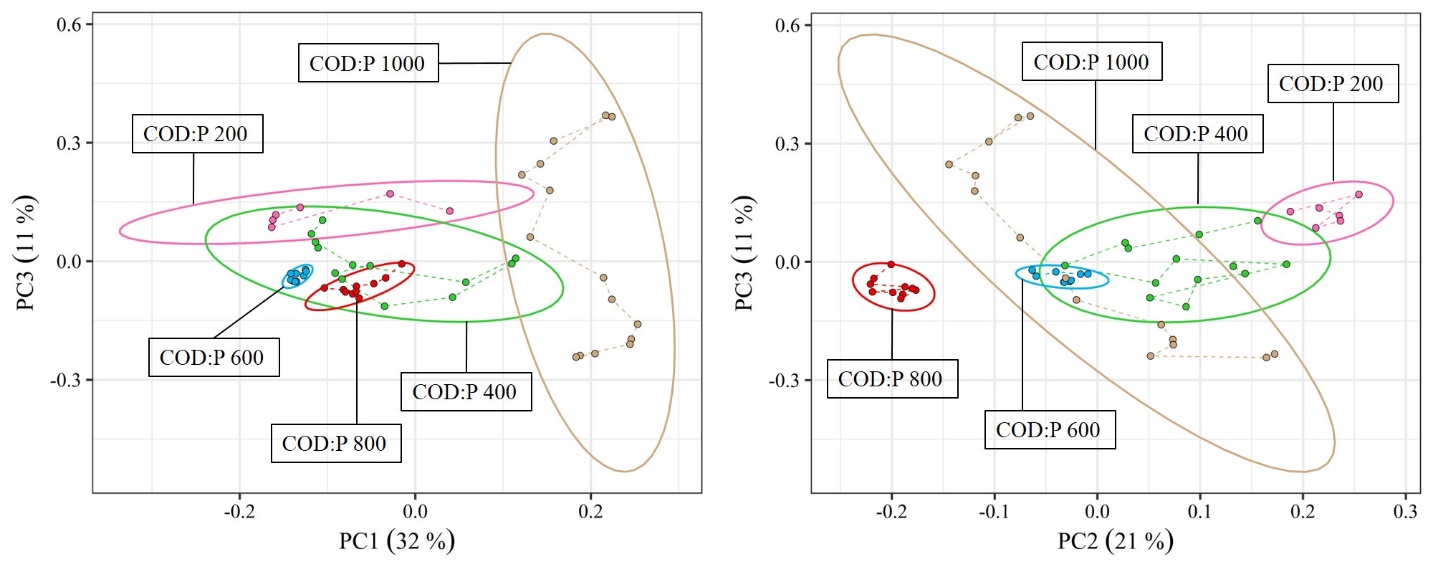  SI Figure A7: Principal Component Analysis (PCA) plots based on Hellinger-transformed relative abundances of the bacterial taxa (genus level) present in the different reactors during steady state. PC3 vs PC1 and PC3 vs PC2 plots complementing Figure 2B of the main manuscript. |
| --- |

## Phosphorus content of the PHA-free biomass (Biomass iP)

| 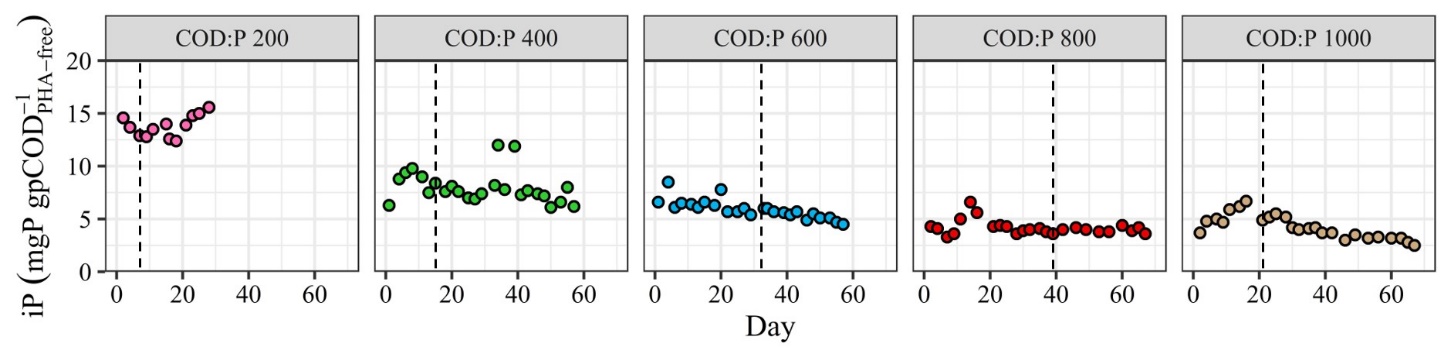  SI Figure A8: Biomass iP over time in the different reactors. The vertical dashed line indicates the onset of steady-state with regard to the microbial community composition. |
| --- |

## Biomass PHA content

| 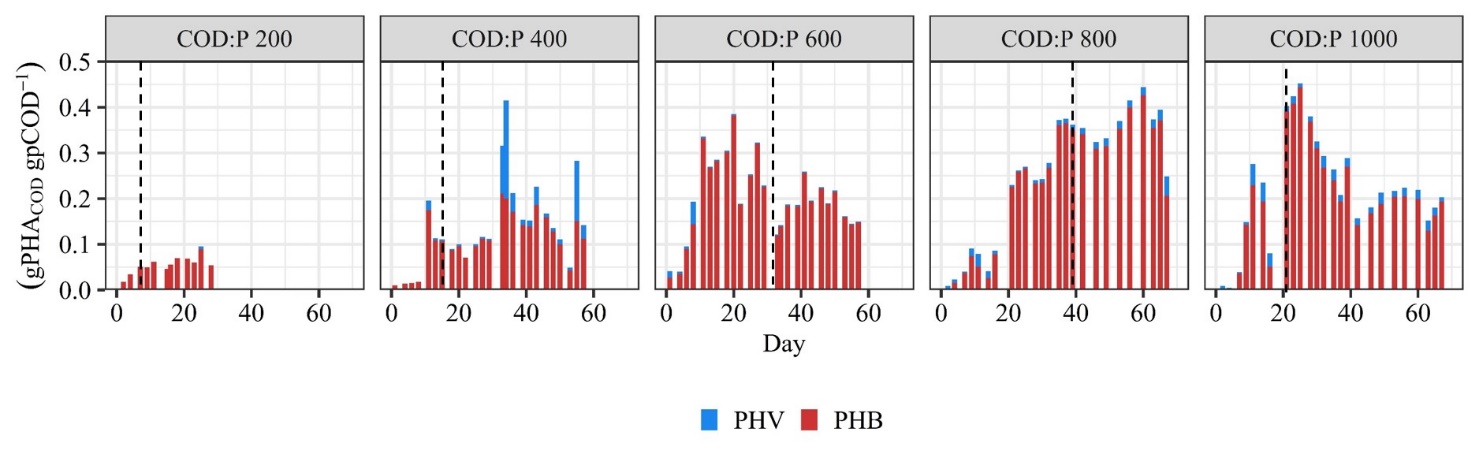  SI Figure A9: PHA-content and monomeric composition over time in the different reactors. The vertical dashed line indicates the onset of steady-state with regard to the microbial community composition. |
| --- |

## Removal performances

| 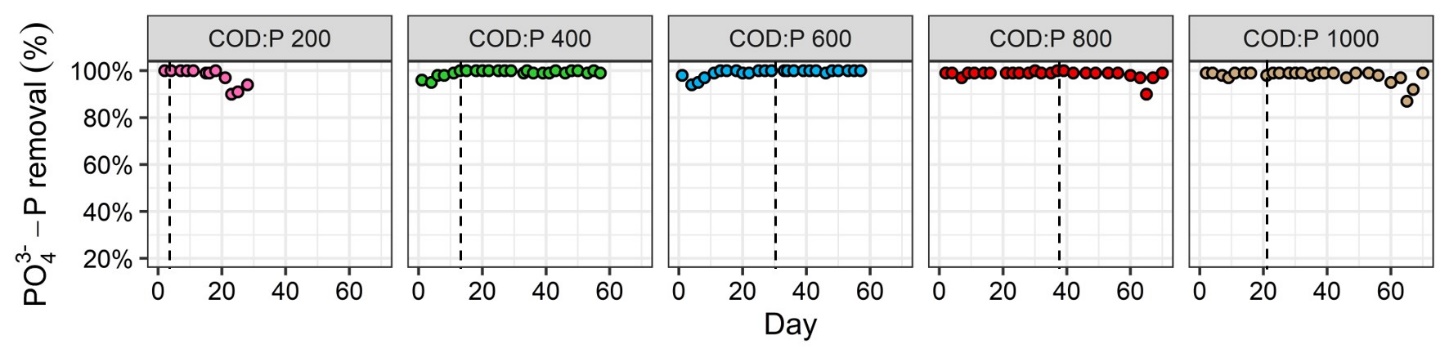  SI Figure A10: PO_4_^3—^P-removal over time in the different reactors. The dashed line indicates the onset of steady-state with regard to the microbial community composition. |
| --- |

## Relationship between influent COD:P ratio, biomass iP and microbial diversity

| 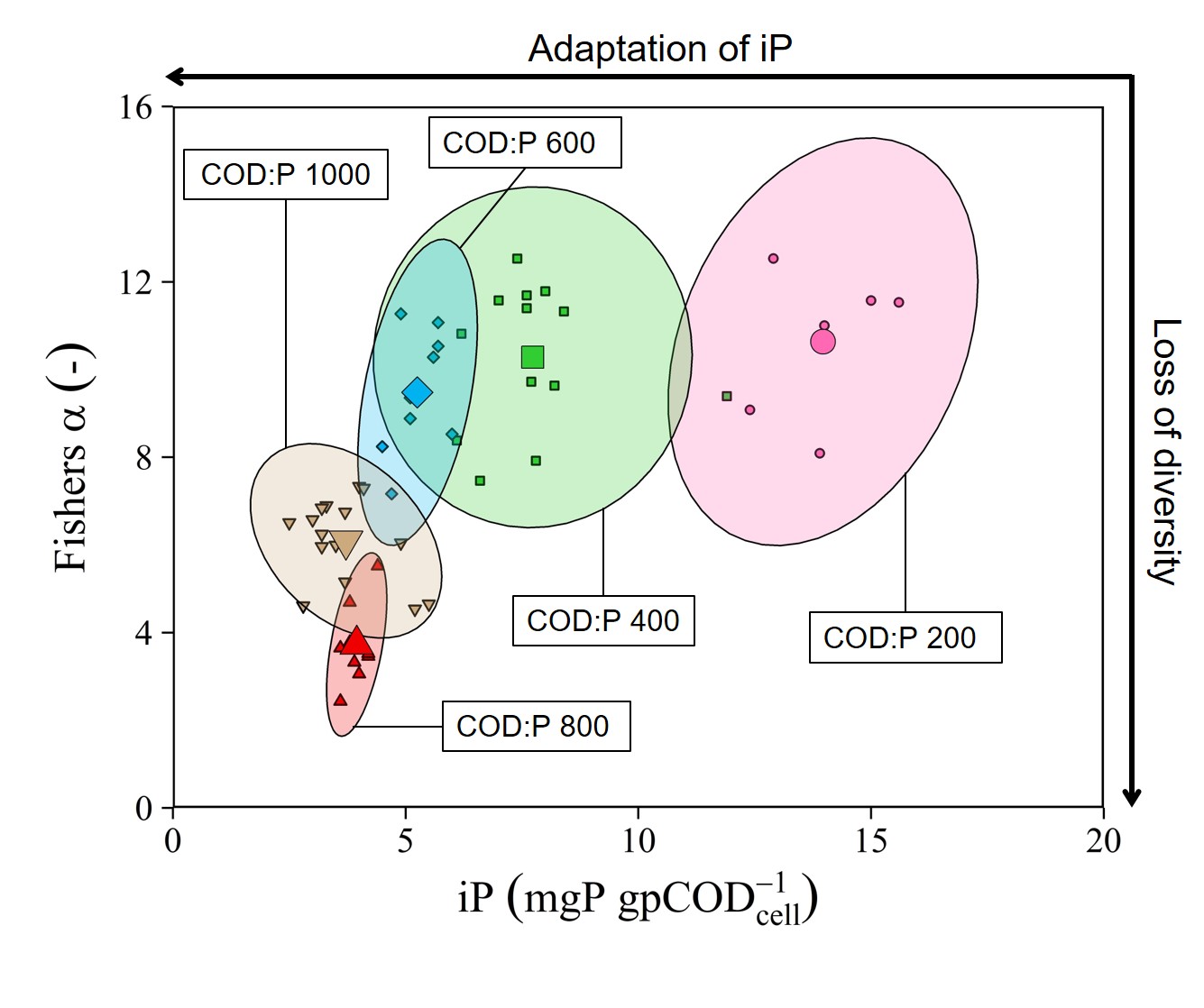  SI Figure A11: Scatter-plot showing the Fischer’s alpha diversity index as a function of the biomass iP in the different reactors. The large symbols represent the average values computed from the individual measurements (small symbols). The ellipses cover the 90% confidence interval, assuming a normal distribution. For this plot, only measurements during steady-state were considered. |
| --- |

# Discussion

**SI Table A8:** Relative abundance of SBR-typical PHA-storers (Paracoccus and Thauera) as opposed to Pannonibacter and Xanthobacter in the inoculum of our different reactors.

|  | **Relative abundance in the inoculum (%)** | | | | |
| --- | --- | --- | --- | --- | --- |
| **Genus** | **COD:P 200** | **COD:P 400** | **COD:P 600** | **COD:P 800** | **COD:P 1000** |
| *Paracoccus* | 0.202 | 0.133 | 0.263 | 0.281 | 0.114 |
| *Thauera* | 0.178 | 0.207 | 0.181 | 0.481 | 0.343 |
| *Pannonibacter* | 0.004 | 0.012 | 0.010 | 0.017 | 0.032 |
| *Xanthobacter* | 0.012 | 0.017 | 0.005 | 0.003 | 0.016 |

**References**

Borsodi, A.K., Micsinai, A., Kovacs, G., Toth, E., Schumann, P., Kovacs, A.L., Boddi, B. and Marialigeti, K. (2003) Pannonibacter phragmitetus gen. nov., sp nov., a novel alkalitolerant bacterium isolated from decomposing reed rhizomes in a Hungarian soda lake. International Journal of Systematic and Evolutionary Microbiology 53, 555-561.

Clifton‐García, B., González‐Reynoso, O., Robledo‐Ortiz, J.R., Villafaña‐Rojas, J. and González‐García, Y. (2020) Forest soil bacteria able to produce homo and copolymers of polyhydroxyalkanoates from several pure and waste carbon sources. Letters in Applied Microbiology 70(4), 300-309.

Ferreira, A.M., Queirós, D., Gagliano, M.C., Serafim, L.S. and Rossetti, S. (2016) Polyhydroxyalkanoates-accumulating bacteria isolated from activated sludge acclimatized to hardwood sulphite spent liquor. Annals of Microbiology 66(2), 833-842.

Gasser, I., Mã¼Ller, H. and Berg, G. (2009) Ecology and characterization of polyhydroxyalkanoate-producing microorganisms on and in plants. FEMS Microbiology Ecology 70(1), 142-150.

Holmes, B., Owen, R.J. and Hollis, D.G. (1982) Flavobacterium-Spiritivorum, a New Species Isolated from Human Clinical Specimens. International Journal of Systematic Bacteriology 32(2), 157-165.

Imhoff, J.F. (2015) Bergey's Manual of Systematics of Archaea and Bacteria, pp. 1-12.

Kumar, V., Thakur, V., Ambika, Kumar, S. and Singh, D. (2018) Bioplastic reservoir of diverse bacterial communities revealed along altitude gradient of Pangi-Chamba trans-Himalayan region. FEMS Microbiology Letters 365(14).

Layer, M., Adler, A., Reynaert, E., Hernandez, A., Pagni, M., Morgenroth, E., Holliger, C. and Derlon, N. (2019) Organic substrate diffusibility governs microbial community composition, nutrient removal performance and kinetics of granulation of aerobic granular sludge. Water Research X 4.

Li, M. and Wilkins, M.R. (2020) Recent advances in polyhydroxyalkanoate production: Feedstocks, strains and process developments. International Journal of Biological Macromolecules 156, 691-703.

Liu, C., Wang, H., Xing, W. and Wei, L. (2013) Composition diversity and nutrition conditions for accumulation of polyhydroxyalkanoate (PHA) in a bacterial community from activated sludge. Applied Microbiology and Biotechnology 97(21), 9377-9387.

Liu, S., Chen, Q., Ma, T., Wang, M. and Ni, J. (2018) Genomic insights into metabolic potentials of two simultaneous aerobic denitrification and phosphorus removal bacteria, Achromobacter sp. GAD3 and Agrobacterium sp. LAD9. FEMS Microbiology Ecology 94(4).

Marín, I. and Arahal, D.R. (2014), pp. 115-133, Springer Berlin Heidelberg.

Martinez, V., de la Pena, F., Garcia-Hidalgo, J., de la Mata, I., Garcia, J.L. and Prieto, M.A. (2012) Identification and Biochemical Evidence of a Medium-Chain-Length Polyhydroxyalkanoate Depolymerase in the Bdellovibrio bacteriovorus Predatory Hydrolytic Arsenal. Applied and Environmental Microbiology 78(17), 6017-6026.

Ray, S., Prajapati, V., Patel, K. and Trivedi, U. (2016) Optimization and characterization of PHA from isolate Pannonibacter phragmitetus ERC8 using glycerol waste. International Journal of Biological Macromolecules 86, 741-749.

Sheu, S.-Y., Sheu, D.-S., Sheu, F.-S. and Chen, W.-M. (2013a) Gemmobacter tilapiae sp. nov., a poly-β-hydroxybutyrate-accumulating bacterium isolated from a freshwater pond. International Journal of Systematic and Evolutionary Microbiology 63(Pt_4), 1550-1556.

Sheu, S.-Y., Shiau, Y.-W., Wei, Y.-T. and Chen, W.-M. (2013b) Gemmobacter lanyuensis sp. nov., isolated from a freshwater spring. International Journal of Systematic and Evolutionary Microbiology 63(Pt_11), 4039-4045.

Sood, U., Hira, P., Singh, P., Singh, D.N. and Lal, R. (2022) Bergey's Manual of Systematics of Archaea and Bacteria, pp. 1-84.

Tyagi, P. and Sharma, A. (2021) Utilization of crude paper industry effluent for Polyhydroxyalkanoate (PHA) production. Environmental Technology & Innovation 23.

Von Tigerstrom, R.G. and Stelmaschuk, S. (1985) Localization of the Cell-associated Phosphatase in Lysobacter enzymogenes. Microbiology 131(7), 1611-1618.

Wiegel, J.K.W. (2015) Xanthobacter. Bergey's Manual of Systematics of Archaea and Bacteria, 1-22.

Xi, L.J., Qiao, N.H., Liu, D.J., Li, J., Zhang, J.J. and Liu, J.G. (2018) Pannonibacter carbonis sp nov., isolated from coal mine water. International Journal of Systematic and Evolutionary Microbiology 68(6), 2042-2047.

Xin, Y.H., Zhou, Y.G. and Chen, W.X. (2006) Ancylobacter polymorphus sp. nov. and Ancylobacter vacuolatus sp. nov. International Journal of Systematic and Evolutionary Microbiology 56(6), 1185-1188.

Xin, Y.H., Zhou, Y.G., Zhou, H.L. and Chen, W.X. (2004) Ancylobacter rudongensis sp. nov., isolated from roots of Spartina anglica. International Journal of Systematic and Evolutionary Microbiology 54(2), 385-388.
